# Association of Chorioamnionitis With Bronchopulmonary Dysplasia Among Preterm Infants: A Systematic Review, Meta-analysis, and Metaregression

**DOI:** 10.1001/jamanetworkopen.2019.14611

**Published:** 2019-11-06

**Authors:** Eduardo Villamor-Martinez, María Álvarez-Fuente, Amro M. T. Ghazi, Pieter Degraeuwe, Luc J. I. Zimmermann, Boris W. Kramer, Eduardo Villamor

**Affiliations:** 1 Department of Pediatrics, School for Oncology and Developmental Biology, Maastricht University Medical Center, Maastricht, the Netherlands; 2 Pediatric Cardiology Department, Hospital Ramón y Cajal, Madrid, Spain

## Abstract

**Importance:**

Bronchopulmonary dysplasia (BPD), a chronic lung disease of prematurity, remains one of the major and most common complications of very preterm birth. Insight into factors associated with the pathogenesis of BPD is key to improving its prevention and treatment.

**Objective:**

To perform a systematic review, meta-analysis, and metaregression of clinical studies exploring the association between chorioamnionitis (CA) and BPD in preterm infants.

**Data Sources:**

PubMed and Embase were searched without language restriction (last search, October 1, 2018). Key search terms included *bronchopulmonary dysplasia*, *chorioamnionitis,* and *risk factors*.

**Study Selection:**

Included studies were peer-reviewed studies examining preterm (<37 weeks’ gestation) or very low-birth-weight (<1500 g) infants and reporting primary data that could be used to measure the association between exposure to CA and the development of BPD.

**Data Extraction and Synthesis:**

The Meta-analysis of Observational Studies in Epidemiology (MOOSE) guideline was followed. Data were independently extracted by 2 researchers. A random-effects model was used to calculate odds ratios (ORs) and 95% CIs. Heterogeneity in effect size across studies was studied using multivariate, random-effects metaregression analysis.

**Main Outcomes and Measures:**

The primary outcome was BPD, defined as supplemental oxygen requirement on postnatal day 28 (BPD28) or at the postmenstrual age of 36 weeks (BPD36). Covariates considered as potential confounders included differences between CA-exposed and CA-unexposed infants in gestational age, rates of respiratory distress syndrome (RDS), exposure to antenatal corticosteroids, and rates of early- and late-onset sepsis.

**Results:**

A total of 3170 potentially relevant studies were found, of which 158 met the inclusion criteria (244 096 preterm infants, 20 971 CA cases, and 24 335 BPD cases). Meta-analysis showed that CA exposure was significantly associated with BPD28 (65 studies; OR, 2.32; 95% CI, 1.88-2.86; *P* < .001; heterogeneity: *I*^2^ = 84%; *P* < .001) and BPD36 (108 studies; OR, 1.29; 95% CI, 1.17-1.42; *P* < .001; heterogeneity: *I*^2^ = 63%; *P* < .001). The association between CA and BPD remained significant for both clinical and histologic CA. In addition, significant differences were found between CA-exposed and CA-unexposed infants in gestational age, birth weight, odds of being small for gestational age, exposure to antenatal corticosteroids, and early- and late-onset sepsis. Chorioamnionitis was not significantly associated with RDS (48 studies; OR, 1.10; 95% CI, 0.92-1.34; *P* = .24; heterogeneity: *I*^2^ = 90%; *P* < .001), but multivariate metaregression analysis with backward elimination revealed that a model combining the difference in gestational age and the odds of RDS was associated with 64% of the variance in the association between CA and BPD36 across studies.

**Conclusions and Relevance:**

The results of this study confirm that among preterm infants, exposure to CA is associated with a higher risk of developing BPD, but this association may be modulated by gestational age and risk of RDS.

## Key Points

**Question:**

Is chorioamnionitis a risk factor for developing bronchopulmonary dysplasia in preterm infants?

**Findings:**

This systematic review, meta-analysis, and metaregression found that chorioamnionitis was associated with an increased risk of bronchopulmonary dysplasia in preterm infants but also found significant differences in baseline characteristics between infants with and infants without chorioamnionitis. A multivariate metaregression model combining the difference in gestational age and the odds of respiratory distress syndrome was associated with 64% of the variance in the association between chorioamnionitis and bronchopulmonary dysplasia.

**Meaning:**

Exposure to chorioamnionitis is associated with a higher risk of developing bronchopulmonary dysplasia in preterm infants, but this association may be modulated by gestational age and risk of respiratory distress syndrome.

## Teaser

This systematic review, meta-analysis, and metaregression of clinical studies explores the association between chorioamnionitis and bronchopulmonary dysplasia in preterm infants.

## Introduction

Bronchopulmonary dysplasia (BPD), a chronic lung disease of prematurity, remains one of the major and most common complications of very preterm birth.^1,2,3,4,5,6,7^ The degree of prematurity is the most important predisposing risk factor for BPD, but inflammatory and/or infectious events are suggested to play a key role in the initiation, progression, and severity of BPD.^3,4,5,8,9,10,11,12,13^ The pulmonary inflammatory response may have been initiated in utero, in the setting of chorioamnionitis (CA).^3,4,5,8,9,10^

Besides the aforementioned detrimental effects, clinical observations support the concept that fetal exposure to infection or inflammation may also be beneficial to the very preterm lung.^4,14,15^ Watterberg et al^15^ were the first to report that CA was associated with an increased risk for BPD but a reduced risk for respiratory distress syndrome (RDS). This observation led to the hypothesis that CA exposure accelerated functional lung maturation but increased the vulnerability of the preterm lung to postnatal injury.^4,14,16^ However, the data supporting this hypothesis are inconsistent, and subsequent studies during the past 20 years have found that CA was associated with increased, decreased, or no risk of either BPD or RDS.^4,14,16^

The role of CA as a potential pathogenic factor for BPD has already been the subject of a systematic review and meta-analysis. Hartling et al^10^ included 59 studies (15 295 preterm infants). They found in unadjusted analyses that CA was significantly associated with BPD (odds ratio [OR], 1.89; 95% CI, 1.56-2.30). They found substantial statistical heterogeneity and evidence of publication bias. They also observed that infants exposed to CA had a significantly lower gestational age and birth weight than infants who were not exposed to CA. Moreover, studies adjusting for important confounders (including gestational age and/or birth weight) showed more conservative measures of association between CA and BPD. They concluded that “despite a large body of evidence, CA cannot be definitely considered a risk factor for BPD.”^10^^(pF8)^

After the publication of the meta-analysis by Hartling et al,^10^ many more studies assessing the association between CA and BPD have been published. Some of these studies are of high methodological quality and included a large number of infants. Therefore, in the present study, we aimed to update and expand the meta-analysis of Hartling et al.^10^ In addition, we investigated not only the association between CA and BPD but also the association between CA and RDS and how these 2 associations correlate. We also analyzed the role of potential confounders or intermediate factors, such as gestational age, birth weight, the presence of fetal inflammatory response (ie, funisitis), exposure to antenatal corticosteroids, sepsis, or patent ductus arteriosus, in the association between CA and BPD.

## Methods

We based the method for this systematic review, meta-analysis, and metaregression on earlier meta-analyses on the associations between CA and patent ductus arteriosus,^17^ CA and retinopathy of prematurity,^18^ and CA and intraventricular hemorrhage.^19^ The study was conducted according to the Meta-analysis of Observational Studies in Epidemiology (MOOSE) reporting guideline^20^ and the Preferred Reporting Items for Systematic Reviews and Meta-analyses (PRISMA) reporting guideline.^21^ A protocol was developed prospectively that detailed the specific objectives, criteria for study selection, approach to assessing study quality, clinical outcomes, and statistical methods. The study is reported according to the PRISMA checklist.

### Sources and Search Strategy

A comprehensive literature search was undertaken using the PubMed/MEDLINE and Embase databases from their inception to October 1, 2018. The search terms involved various combinations of the following key words: *chorioamnionitis*, *intrauterine infection*, *intrauterine inflammation*, *antenatal infection*, *antenatal inflammation*, *bronchopulmonary dysplasia*, *chronic lung disease*, *risk factors*, *outcome*, *cohort*, and *case-control*. No language limit was applied. Narrative reviews, systematic reviews, case reports, letters, editorials, and commentaries were excluded but were read to identify potential additional studies. Additional strategies to identify studies included a manual review of reference lists from key articles that fulfilled our eligibility criteria and other systematic reviews on CA, use of the “related articles” feature in PubMed, and use of the “cited by” tool in Web of Science and Google Scholar.

### Study Selection

Studies were included if they examined preterm (gestational age <37 weeks) or very low-birth-weight (<1500 g) infants and reported primary data that could be used to measure the association between exposure to CA and the development of BPD. Therefore, we selected studies assessing the outcomes of infants exposed to CA when BPD was one of the reported outcomes and studies assessing the risk factors for BPD when CA was one of the reported risk factors. To identify relevant studies, 2 of us (A.M.T.G. and E.V.) independently screened the results of the searches and applied inclusion criteria using a structured form. Discrepancies were resolved through discussion or consultation with a third reviewer (P.D.).

### Data Extraction

Two of us (A.M.T.G. and P.D.) extracted data from relevant studies using a predetermined data extraction form, and 3 of us (E.V.-M., M.A.-F., and E.V.) checked data extraction for accuracy and completeness. Discrepancies were resolved by consulting the primary report. Data extracted from each study included citation information, language of publication, location where research was conducted, time period of the study, study objectives, study design, definitions of CA and BPD, inclusion and exclusion criteria, patient characteristics, and results (including raw numbers, summary statistics, and adjusted analyses on CA and BPD when available). Studies that did not define CA were assumed to use a clinical definition. Bronchopulmonary dysplasia defined as supplemental oxygen requirement on postnatal day 28 was coded as *BPD28*. Bronchopulmonary dysplasia defined as oxygen requirement at the postmenstrual age (PMA) of 36 weeks (with or without physiological challenge of supplemental oxygen withdrawal) was coded as *BPD36*. Using these definition criteria, BPD28 was considered to include all severities of BPD, whereas BPD36 was considered to include a combination of moderate and severe BPD.^2^ Data on separate categories of BPD (mild, moderate, and severe) were collected when available.

### Quality Assessment

Methodological quality was assessed using the Newcastle-Ottawa Scale for cohort or case-control studies.^22^ This scale uses a rating system (range, 0-9 points; higher scores indicate reduced bias) that scores 3 aspects of a study: selection (0-4 points), comparability (0-2 points), and exposure or outcome (0-3 points). Studies were evaluated as though the association between CA and BPD was the primary outcome. Two of us (E.V.-M. and E.V.) independently assessed the methodological quality of each study. Discrepancies were resolved through discussion.

### Statistical Analysis

Studies were combined and analyzed using Comprehensive Meta-Analysis, version 3.0 software (Biostat Inc). For dichotomous outcomes, the OR with 95% CI was calculated from the data provided in the studies. Odds ratios adjusted for potential confounders were extracted from the studies reporting these data. For continuous outcomes, the mean difference with 95% CI was calculated. When studies reported continuous variables as median and range or interquartile range, we estimated the mean and SD using the method of Wan et al^23^ and the calculator they provided.^24^

Owing to anticipated heterogeneity, summary statistics were calculated with a random-effects model. This model accounts for variability between studies as well as within studies. Subgroup analyses were conducted according to the mixed-effects model.^25^ In this model, a random-effects model is used to combine studies within each subgroup, and a fixed-effect model is used to combine subgroups and yield the overall effect. The study-to-study variance (τ^2^) is not assumed to be the same for all subgroups. This value is computed within subgroups and not pooled across subgroups. Statistical heterogeneity was assessed by use of the Cochran *Q* statistic and by use of the *I*^2^ statistic, which is derived from the *Q* statistic and describes the proportion of total variation that is due to heterogeneity beyond chance.^26^ We used the Egger regression test and funnel plots to assess publication bias.

To explore differences between studies that might be expected to influence the effect size, we performed random effects (method of moments) univariate and multivariate metaregression analyses.^27^ The potential sources of variability defined a priori were CA type (clinical or histologic), differences in gestational age and birth weight between infants with and infants without CA, use of antenatal corticosteroids, mode of delivery, rate of small-for-gestational-age infants, rate of premature rupture of membranes, rate of preeclampsia, rate of early-onset sepsis, rate of late-onset sepsis, rate of RDS, and mortality. Covariates were selected for further modeling if they significantly (*P* < .05) modified the association between CA and BPD. Subsequently, preselected covariates were included in a backward multiple metaregression analysis with *P* = .05 as a cutoff point for removal. *P* < .05 (*P* < .10 for heterogeneity) was considered statistically significant. All tests were 2-tailed.

## Results

### Description of Studies

**Figure 1.**
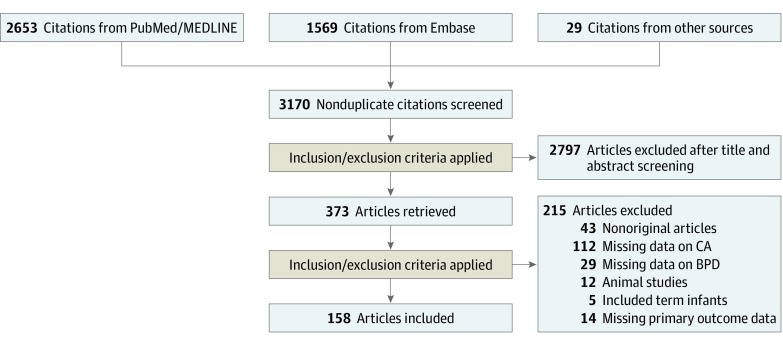
PRISMA Flowchart of the Systematic Search BPD indicates bronchopulmonary dysplasia; and CA, chorioamnionitis.

Of 3170 potentially relevant studies, 158 (5.0%)^13,15,28,29,30,31,32,33,34,35,36,37,38,39,40,41,42,43,44,45,46,47,48,49,50,51,52,53,54,55,56,57,58,59,60,61,62,63,64,65,66,67,68,69,70,71,72,73,74,75,76,77,78,79,80,81,82,83,84,85,86,87,88,89,90,91,92,93,94,95,96,97,98,99,100,101,102,103,104,105,106,107,108,109,110,111,112,113,114,115,116,117,118,119,120,121,122,123,124,125,126,127,128,129,130,131,132,133,134,135,136,137,138,139,140,141,142,143,144,145,146,147,148,149,150,151,152,153,154,155,156,157,158,159,160,161,162,163,164,165,166,167,168,169,170,171,172,173,174,175,176,177,178,179,180,181,182,183^ met the inclusion criteria. The PRISMA flow diagram of the search process is shown in Figure 1. The included studies evaluated 244 096 preterm infants and included 20 791 CA cases and 24 335 cases of BPD of any severity. The included studies and their characteristics are summarized in eTable 1 in the Supplement. Seventy-six studies^15,28,29,30,31,32,33,34,35,36,37,38,39,40,41,42,43,44,45,46,47,48,49,50,51,52,53,54,55,56,57,58,59,60,61,62,63,64,65,66,67,68,69,70,71,72,73,74,75,76,77,78,79,80,81,82,83,84,85,86,87,88,89,90,91,92,93,94,95,96,97,98,99,100,101,183^ were designed from the perspective of CA; they examined the outcomes of preterm infants with or without CA, and BPD was one of these outcomes. Sixty-seven studies^100,102,103,104,105,106,107,108,109,110,111,112,113,114,115,116,117,118,119,120,121,122,123,124,125,126,127,128,129,130,131,132,133,134,135,136,137,138,139,140,141,142,143,144,145,146,147,148,149,150,151,152,153,154,155,156,157,158,159,160,161,162,163,164,165,166,167^ were designed from the perspective of BPD; they studied risk factors for BPD, and CA was one of these risk factors. Sixteen studies^13,168,169,170,171,172,173,174,175,176,177,178,179,180,181,182^ were designed to primarily examine the association between CA and BPD. Forty-eight included studies examined the association between CA and RDS.

Forty-two studies defined CA clinically, and 97 studies defined CA histologically. Six studies provided BPD outcomes for infants with histologic and clinical CA separately.^68,75,80,116,127,146^ One study required infants to have both histologic and clinical CA to be considered exposed to CA.^84^ Nine studies defined CA using a microbiological definition.^28,29,47,53,58,64,131,135,152^ Finally, 16 studies^102,104,105,108,113,120,123,128,129,130,139,141,148,155,160,170^ did not define CA and, for the purposes of analysis, were considered to evaluate clinical CA.

Most studies included infants with a gestational age less than 32 weeks or a birth weight less than 1500 g, as described in eTable 1 in the Supplement. Eighty-one studies included infants who were 32 weeks’ gestational age or more preterm, 27 studies included infants who were at most 32 to 34 weeks’ gestational age, and 10 studies included infants who were less than 34 to 37 weeks’ gestational age. Nine studies included infants who had a birth weight of less than 1000 g, 49 studies included infants who had a birth weight of 1500 g or less, and 2 studies included infants who had a birth weight of 2000 g or less. Finally, 4 studies used inclusion criteria (clarified per study in eTable 1 in the Supplement) other than gestational age or birth weight.

Sixty-five studies provided data on BPD28, and 108 studies provided data on BPD36. Fifteen studies provided data on the incidence of mild BPD, 7 studies provided data on the incidence of moderate BPD, and 8 studies provided data on the incidence of severe BPD.

### Analysis Based on Unadjusted Data

**Figure 2.**
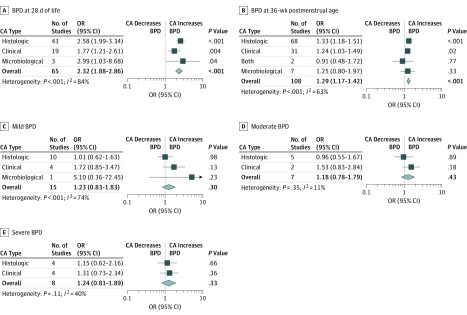
Meta-analyses of the Association Between Chorioamnionitis (CA) and Bronchopulmonary Dysplasia (BPD) Grouped by definition of CA. OR indicates odds ratio.

Meta-analysis found a positive association between exposure to CA and BPD28 (65 studies; OR, 2.32; 95% CI, 1.88-2.86; *P* < .001; heterogeneity: *I*^2^ = 84%; *P* < .001) (Figure 2A). When subdividing by definition of CA, we found that the association with BPD28 remained significant for histologic CA (OR, 2.58; 95% CI, 1.99-3.34), clinical CA (OR 1.77, 95% CI, 1.21-2.61), and microbiological CA (OR, 2.99; 95% CI, 1.03-8.68) (Figure 2A; eFigure 1 in the Supplement). We also found a significant positive association between CA and BPD36 (108 studies; OR, 1.29; 95% CI, 1.17-1.42; *P* < .001; heterogeneity: *I*^2^ = 63%; *P* < .001) (Figure 2B). This association was also significant when pooling only studies of histologic CA (OR, 1.33; 95% CI, 1.18-1.51) (Figure 2B; eFigure 2 in the Supplement) and clinical CA (OR, 1.24; 95% CI, 1.03-1.49) (Figure 2B; eFigure 3 in the Supplement).

When further stratified by grade of BPD, meta-analysis did not find a significant association between CA and mild BPD (15 studies; Figure 2C; eFigure 4 in the Supplement), moderate BPD (7 studies; Figure 2D; eFigure 5 in the Supplement), or severe BPD (8 studies; Figure 2E; eFigure 6 in the Supplement). Twenty-three of the 158 included studies also reported on funisitis and risk of BPD. Meta-analysis did not find a difference between infants exposed to CA with funisitis and infants exposed to CA without funisitis in the risk of BPD28 (OR, 1.26; 95% CI, 0.61-2.59) or the risk of BPD36 (OR, 1.19; 95% CI, 0.77-1.83) (eFigure 7 in the Supplement).

### Analysis of Adjusted Data

To examine confounding factors, we pooled studies that provided adjusted data on the association between CA and BPD. Eleven studies reported adjusted data on BPD28. Meta-analysis of these adjusted data showed a significant association between CA and BPD28 (OR, 1.68; 95% CI, 1.28-2.21) (eFigure 8 in the Supplement). When the unadjusted data on BPD28 from the 11 studies were pooled, the OR increased to 2.17 (95% CI, 1.71-2.76). However, metaregression did not find this increase in effect size to be statistically significant (*P* = .17).

Twenty-one studies reported adjusted data on BPD36. Meta-analysis of these adjusted data showed a significant association between CA and BPD36 (OR, 1.25; 95% CI, 1.01-1.54) (eFigure 9 in the Supplement). When the unadjusted data on BPD36 of the 21 studies were pooled, the OR increased to 1.65 (95% CI, 1.37-2.00). Metaregression did not find this increase in effect size to be statistically significant (*P* = .05).

### Analysis of Covariates and Metaregression

**Figure 3.**
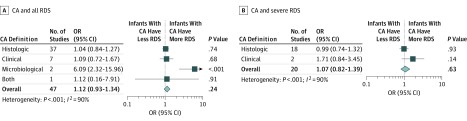
Meta-analysis of the Association Between Chorioamnionitis (CA) and Respiratory Distress Syndrome (RDS) Grouped by definition of CA and severity of RDS. OR indicates odds ratio.

We performed additional meta-analyses to explore the possible differences in baseline characteristics between the groups exposed or nonexposed to CA. As summarized in the Table, infants exposed to CA showed a significantly lower gestational age (difference in means, –1.20 weeks; 95% CI, –1.48 to –0.92 weeks) and birth weight (difference in means, –48 g; 95% CI, –66 to –30 g) and significantly lower rates of birth by cesarean delivery (OR, 0.35; 95% CI, 0.28-0.43), small for gestational age (OR, 0.34; 95% CI, 0.26-0.44), and preeclampsia (OR, 0.16; 95% CI, 0.11-0.23). Moreover, infants exposed to CA showed significantly higher rates of exposure to antenatal corticosteroids (OR, 1.39; 95% CI, 0.98-1.97), premature rupture of membranes (OR, 3.66; 95% CI, 3.02-4.44), early-onset sepsis (OR, 3.18; 95% CI, 2.41-4.19), late-onset sepsis (OR, 1.32; 95% CI, 1.10-1.58), and mortality (OR, 1.48; 95% CI, 1.28-1.71). In contrast, meta-analysis did not demonstrate a significant association between CA and all RDS (OR, 1.10; 95% CI, 0.92-1.34; *P* = .24; heterogeneity: *I*^2^ = 90%; *P* < .001) (Figure 3; eFigure 10 in the Supplement) or severe RDS (defined by the necessity of surfactant and/or mechanical ventilation) (Figure 3; eFigure 11 in the Supplement).

**Table.** Meta-analyses of the Association Between Exposure to Chorioamnionitis and Baseline Characteristics and Outcomes^a^

Meta-analysis	Studies, No.	Effect Size, OR (95% CI)	*P* Value	Heterogeneity	
				*I*^2^, %	*P* Value
Gestational age, wk	65	−1.20 (−1.48 to −0.92)^b^	<.001	98	<.001
Birth weight, g	64	−48 (−66 to −30)^b^	<.001	94	<.001
Antenatal corticosteroids	53	1.39 (0.98 to 1.97)	<.001	97	<.001
Cesarean delivery	42	0.35 (0.28 to 0.43)	<.001	92	<.001
Small for gestational age	24	0.34 (0.26 to 0.44)	<.001	77	<.001
Preeclampsia	19	0.16 (0.11 to 0.23)	<.001	78	<.001
Premature rupture of membranes	37	3.66 (3.02 to 4.44)	<.001	86	<.001
Maternal diabetes	10	0.85 (0.68 to 1.05)	.13	3	.41
Maternal age, y	26	−0.14 (−0.47 to 0.19)^b^	.41	61	<.001
Sepsis onset					
Early	34	3.18 (2.41 to 4.19)	<.001	79	<.001
Late	33	1.32 (1.10 to 1.58)	.003	75	<.001
Respiratory distress syndrome					
All	48	1.12 (0.93 to 1.34)	.24	90	<.001
Severe	20	1.07 (0.82 to 1.39)	.63	90	<.001
Mortality	42	1.48 (1.28 to 1.71)	<.001	61	<.001

Abbreviation: OR, odds ratio.

^a^ The group of infants with chorioamnionitis is considered the study group for differences in mean values and ORs; for example, infants with chorioamnionitis were born earlier (difference, −1.20 weeks) than infants without chorioamnionitis, and infants with chorioamnionitis had a lower rate of cesarean delivery (OR, 0.35) than infants without chorioamnionitis.

^b^ Differences in mean values.

**Figure 4.**
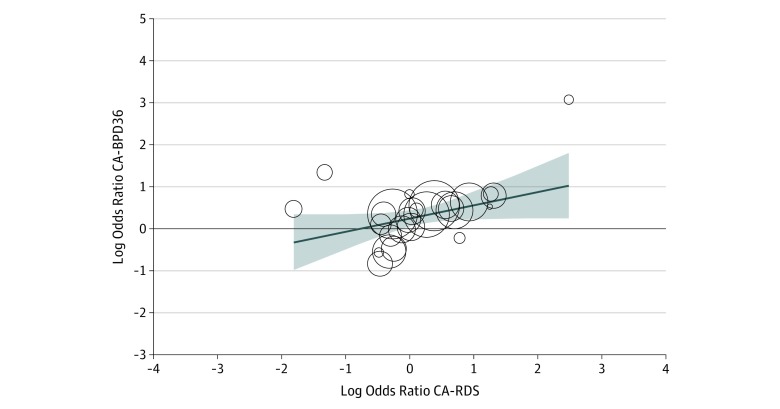
Multivariate Metaregression Analysis of Chorioamnionitis (CA) and Bronchopulmonary Dysplasia (BPD) and CA and Respiratory Distress Syndrome Multivariate regression model with backward elimination, controlling for difference in gestational age between CA-exposed and CA-unexposed infants. A total of 27 studies were included (coefficient, 0.31; 95% CI, 0.09-0.54; *P* = .007; *R*^2^ equivalent, 0.64). BPD36 indicates BPD with supplemental oxygen requirement at the postmenstrual age of 36 weeks.

Relevant covariates were preselected using univariate metaregression analyses. Results of all univariate analyses are presented in eTable 2 in the Supplement. For BPD28, we found that mean difference in gestational age significantly explained heterogeneity in effect size across studies (eTable 2 in the Supplement). For BPD36, we found that mean difference in gestational age (eFigure 12 and eTable 2 in the Supplement), mean difference in birth weight (eTable 2 in the Supplement), and RDS risk (eFigure 13 and eTable 2 in the Supplement) significantly explained heterogeneity in effect size across studies; these variables were therefore considered for further modeling. Backward multiple metaregression analysis, including all studies on BPD36 with complete data for these 3 covariates (gestational age, birth weight, and RDS; *k* = 25), revealed that heterogeneity in effect size across studies was significantly explained by the mean difference in gestational age (coefficient, –0.23; 95% CI, −0.40 to −0.06; *P* = .008) and risk of RDS (coefficient, 0.31; 95% CI, 0.09-0.54; *P* = .007). We retested this model, including all studies with complete data on mean difference in gestational age and risk of RDS (*k* = 27) (Figure 4). This final model had a total explained variance of 64% (*R*^2^ equivalent). The variance did not seem to be inflated owing to multicollinearity (variance inflation factor = 1.08). Each week that infants with CA are born earlier than control infants resulted in an increase in BPD36 log OR of 0.23 (the equivalent of going from an OR of 1.00 to an OR of 1.70). Each point increase in the RDS log OR resulted in an increase in the BPD36 log OR of 0.31 (the equivalent of going from an OR of 1.00 to an OR of 2.04).

To further assess gestational age as a confounding factor, we performed a meta-analysis of studies in which the mean difference in gestational age was not significant (*P* > .05). As shown in eFigure 14 in the Supplement, we observed no differences in BPD28 risk in studies with similar gestational age (6 studies). On the other hand, when a significant difference in gestational age was observed (*P* < .05), CA was significantly associated with BPD28 (20 studies; OR, 2.00; 95% CI, 1.49-2.69) (eFigure 14 in the Supplement). We found similar results for BPD36. Meta-analysis of studies in which the mean difference in gestational age was not significant did not find an association between CA and BPD36 (15 studies; eFigure 15 in the Supplement), whereas meta-analysis of studies in which the difference in gestational age was significant found a significant association between CA and BPD36 risk (32 studies; OR, 1.43; 95% CI, 1.23-1.67).

### Quality Assessment

The quality of each study according to the Newcastle-Ottawa Scale is summarized in eTable 3 in the Supplement. Studies received a quality score of 6 points (2 studies), 7 points (21 studies), 8 points (112 studies), or 9 points (23 studies), out of a possible 9 points. Studies were downgraded in quality most frequently for not adjusting the risk of BPD for confounders (133 studies), for not defining CA clearly (16 studies), and for not defining BPD precisely (3 studies).

### Publication Bias

Neither visual inspection of funnel plots (eFigure 16 in the Supplement) nor the Egger test suggested publication or selection bias. There was an insufficient number of studies with other BPD definitions (ie, mild, moderate, or severe) to evaluate publication bias.

## Discussion

The present study is a substantial update to the systematic review of Hartling et al,^10^ including a larger number of studies (158 vs 59), a much larger number of infants (244 096 vs 15 295), and a wider range of analysis of covariates. Our study confirms the results of Hartling et al^10^ and adds new information on the role of funisitis and RDS, which have not been previously systematically reviewed, to our knowledge. Chorioamnionitis was a significant risk factor for BPD28 (all BPD) and for BPD36 (moderate and severe BPD), but a significant association with severe BPD was not demonstrated. Exposure to funisitis was not significantly associated with a higher risk of BPD compared with exposure to CA in the absence of funisitis. Meta-analysis did not demonstrate a significant association between CA and RDS. As in earlier meta-analyses of CA and morbidities,^17,184,185^ we found significant differences between CA-exposed and CA-unexposed infants in gestational age, birth weight, odds of being small for gestational age, exposure to antenatal corticosteroids, early- and late-onset sepsis, and patent ductus arteriosus. Multivariate metaregression analysis revealed that a model combining the difference in gestational age and the odds of RDS explained 64% of the variance in the association between CA and BPD36 across studies. In conclusion, our results confirm the positive association between CA and BPD in preterm infants, but the pathogenic effect of CA on BPD may be modulated by the effect of CA on gestational age and risk of RDS.

As discussed elsewhere,^186^ one important limitation inherent to any meta-analysis of BPD is the heterogeneity of the definition of the condition.^187,188,189^ The first clinical definition for BPD was adopted as infants requiring supplemental oxygen on postnatal day 28.^5,187^ In 1988, the definition was refined to oxygen use at 36 weeks of PMA,^190^ and 12 years later, a categorization of BPD as mild, moderate, or severe was proposed.^2^ Mild BPD included infants who received oxygen or respiratory support at the postnatal age of 28 days but who were breathing room air at 36 weeks PMA. When infants required supplemental oxygen at 36 weeks PMA, BPD was classified as moderate (need for <30% oxygen) or severe (need for ≥30% oxygen and/or positive airway pressure).^2^ A further refinement in the definition included a physiological challenge of supplemental oxygen withdrawal to test for oxygen need at 36 weeks PMA.^191^ Most of the studies included in our meta-analysis defined BPD using the 36 weeks of PMA criteria (BPD36). Therefore, they provided data on combined moderate and severe BPD. This combination fails to differentiate the infants with more severe BPD, who remain dependent on mechanical ventilation and more often have severe complications, including pulmonary hypertension, poor growth, and neurodevelopmental problems.^6^ Only 7 studies provided separate data on severe BPD. Meta-analysis could not demonstrate a significant association between CA and severe BPD, but the small number of studies is the main limitation of this subanalysis.

Another main difficulty when assessing CA as a risk factor for neonatal adverse outcomes is the absence of a “healthy” control group. Possible causes of very preterm birth (ie, gestational age <32 weeks) can be divided into 2 main categories: infection and/or inflammation and dysfunctional placentation.^192^ Chorioamnionitis is associated with infection and/or inflammation, and we and others have previously found that infants exposed to CA differ substantially from nonexposed infants in relevant clinical characteristics and outcomes.^17,184,185^ We replicated these findings in the present study and found that CA-exposed infants were born earlier (1.2 weeks) and weighed less (48 g) than infants without CA. In addition, they were more frequently exposed to antenatal corticosteroids, they were less frequently small for gestational age, and they had higher rates of early- and late-onset sepsis, as well as a higher mortality rate. We performed metaregression to analyze how these differences between the CA-exposed and the nonexposed infants affected the association between CA and BPD. Univariate metaregression showed that differences in gestational age and birth weight, as well as rate of RDS, significantly modified the CA-associated risk of BPD36. As already mentioned, multivariate regression found that 64% of variance in CA-associated BPD risk was explained by the differences in gestational age and rate of RDS.

The so-called Waterberg hypothesis or early-protection, late-damage effect suggests that CA may be associated with a reduction in RDS but an increase in BPD.^4,14,16^ To test this hypothesis, we also analyzed the association between CA and RDS in the included studies. In contrast to BPD, meta-analysis of unadjusted data could not demonstrate a significant association between CA and the development of RDS. Prematurity is the most important risk factor for RDS. The CA-exposed infants were born 1.2 weeks earlier but did not show a higher rate of RDS. This finding may suggest some degree of protection against RDS, compatible with the Waterberg hypothesis. In contrast, metaregression showed a significant positive association between the effect size of the CA-RDS association and the effect size of the CA-BPD association (eFigure 12 in the Supplement). In other words, the studies showing a higher risk of RDS in the CA group also showed a higher risk of BPD. Nevertheless, our results should be interpreted with caution because the criteria for the definition of RDS varied substantially among the different studies. As pointed out by Jobe and Kallapur,^193^ although RDS is the diagnosis assigned to most preterm infants, it is unlikely that they have only 1 lung disease at birth. We therefore restrained the analysis to the studies defining a more severe form of RDS (ie, RDS requiring surfactant and/or mechanical ventilation), which, however, did not modify the lack of association between CA and RDS.

It remains unclear whether the most severe grades of CA with a fetal inflammatory response further increase the risk for developing BPD and/or RDS. Funisitis is considered the histologic counterpart of the fetal inflammatory response syndrome.^194,195^ Been et al^34^ showed that the presence of funisitis categorized infants at risk for severe RDS who were less responsive to surfactant treatment. In contrast, infants exposed to CA without funisitis had less severe RDS than did infants without the CA exposure. Therefore, exposure to CA and/or funisitis may be more strongly associated with the severity than with the incidence of respiratory complications. Our meta-analysis did not demonstrate that the presence of funisitis significantly increased the risk of BPD or RDS compared with CA in the absence of funisitis (eFigure 7 in the Supplement). However, our meta-analysis is limited by the small number of studies providing data on funisitis. In addition, infants with funisitis also presented with differences in basal characteristics (including lower gestational age) compared with infants with CA without funisitis.^17,184,185^

### Limitations and Strengths

Hartling et al^10^ noted 2 problems that made the interpretation of their results difficult: significant publication bias and substantial statistical heterogeneity. Our larger study showed a similarly high degree of heterogeneity, but we did not find statistical evidence of publication bias. Egger regression can test only for data trends that may be caused by selective reporting, publication, or inclusion.^196^ Even highly significant data trends do not necessarily mean that the results of the primary analysis are biased.^196^ In addition, the Egger regression test has a high type I error rate and may generate false-positives when dealing with many studies and a high degree of heterogeneity.^197^

Some additional limitations of our systematic review and meta-analysis deserve consideration. First, the published literature showed great heterogeneity in the definition of CA and in the assessment of confounders. In particular, criteria for the use of the term *clinical CA* are highly variable, and recent recommendations propose restricting the term *chorioamnionitis* to pathologic diagnosis.^198^ In addition, the term *funisitis* was not included in our search strategy. Second, only a limited number of studies evaluated the association between CA and BPD as their main objective. Similarly, adjusted data were available only from a subset of all studies included in the meta-analysis. In addition, we had to rely on the adjusted analyses as presented in the published reports and the variables for which they adjusted, which were not consistent across studies. Third, metaregression uses summary data at the study level, which means that we cannot comment on data of individual infants within a study and that there is a risk of ecological bias.^199^ The main strengths of the present study are the large number of studies included and the use of rigorous methods, including duplicate screening, inclusion, and data extraction to reduce bias; meta-analysis of baseline and secondary characteristics; and the use of metaregression to control for potential confounders.

## Conclusions

Overall, the results of this study confirm that, among preterm infants, exposure to CA is associated with a higher risk of developing BPD, but this association may be modulated by gestational age and risk of RDS. Bronchopulmonary dysplasia remains a persistent problem in part because advances in neonatal care have improved the survival of the youngest and smallest infants, who are more prone to develop BPD.^5^ Our data show that CA is frequently the cause of prematurity among these youngest and smallest infants. This higher degree of prematurity may alter the association between CA and BPD. In addition, CA may initiate the pathogenic sequence leading to BPD but also may alter the rate of exposure to other anti-inflammatory or proinflammatory stimuli, such as antenatal corticosteroids, RDS, patent ductus arteriosus, mechanical ventilation, oxygen, and sepsis. Nevertheless, CA, RDS, and BPD are imprecise diagnoses and have been partially changed over time, making the analysis of their associations and correlations difficult.

## Notes

Supplement. **eFigure 1.** Meta-Analysis of the Association Between Chorioamnionitis and All Bronchopulmonary Dysplasia **eFigure 2.** Meta-Analysis of the Association Between Histological Chorioamnionitis and Moderate/Severe Bronchopulmonary Dysplasia **eFigure 3.** Meta-Analysis of the Association Between Different Types of Chorioamnionitis and Moderate/Severe Bronchopulmonary Dysplasia **eFigure 4.** Meta-Analysis of the Association Between Chorioamnionitis and Mild Bronchopulmonary Dysplasia **eFigure 5.** Meta-Analysis of the Association Between Chorioamnionitis and Moderate Bronchopulmonary Dysplasia **eFigure 6.** Meta-Analysis of the Association Between Chorioamnionitis and Severe Bronchopulmonary Dysplasia **eFigure 7.** Meta-Analysis of the Association Between Funisitis and Bronchopulmonary Dysplasia **eFigure 8.** Meta-Analysis of Chorioamnionitis and BPD28, Grouped by Use of Adjusted/Unadjusted Odds Ratios **eFigure 9.** Meta-Analysis of Chorioamnionitis and BPD36, Grouped by Use of Adjusted/Unadjusted Odds Ratios **eFigure 10.** Meta-Analysis of the Association Between Chorioamnionitis and all Respiratory Distress Syndrome **eFigure 11.** Meta-Analysis of the Association Between Chorioamnionitis and Severe RDS **eFigure 12.** Meta-Regression Plot of Association Between Chorioamnionitis and BPD36 Controlling for Difference in Gestational Age **eFigure 13.** Meta-Regression Plot of Association Between Chorioamnionitis and BPD36 Controlling for Risk of RDS **eFigure 14.** Meta-Analysis of Chorioamnionitis and BPD28, Grouped by Difference in Gestational Age **eFigure 15.** Meta-Analysis of Chorioamnionitis and BPD36, Grouped by Significant/Nonsignificant Difference in Gestational Age **eFigure 16.** Funnel Plots Assessing Publication Bias for the Association Between Chorioamnionitis and Bronchopulmonary Dysplasia **eTable 1.** Characteristics of All Included Studies **eTable 2.** Meta-Regression Analyses of Risk of BPD and Covariates **eTable 3.** Newcastle-Ottawa Quality Assessment of Included Studies
