# Supplementary material for: Association of Chorioamnionitis With Bronchopulmonary Dysplasia Among Preterm Infants: A Systematic Review, Meta-analysis, and Metaregression
Source: JAMA Netw Open. 2019 Nov 6;2(11):e1914611. doi: 10.1001/jamanetworkopen.2019.14611 (PMC6865274; doi:10.1001/jamanetworkopen.2019.14611)
Supplement: Supplement. — eFigure 1. Meta-Analysis of the Association Between Chorioamnionitis and All Bronchopulmonary Dysplasia eFigure 2. Meta-Analysis of the Association Between Histological Chorioamnionitis and Moderate/Severe Bronchopulmonary Dysplasia eFigure 3. Meta-Analysis of the Association Between Different Types of Chorioamnionitis and Moderate/Severe Bronchopulmonary Dysplasia eFigure 4. Meta-Analysis of the Association Between Chorioamnionitis and Mild Bronchopulmonary Dysplasia eFigure 5. Meta-Analysis of the Association Between Chorioamnionitis and Moderate Bronchopulmonary Dysplasia eFigure 6. Meta-Analysis of the Association Between Chorioamnionitis and Severe Bronchopulmonary Dysplasia eFigure 7. Meta-Analysis of the Association Between Funisitis and Bronchopulmonary Dysplasia eFigure 8. Meta-Analysis of Chorioamnionitis and BPD28, Grouped by Use of Adjusted/Unadjusted Odds Ratios eFigure 9. Meta-Analysis of Chorioamnionitis and BPD36, Grouped by Use of Adjusted/Unadjusted Odds Ratios eFigure 10. Meta-Analysis of the Association Between Chorioamnionitis and all Respiratory Distress Syndrome eFigure 11. Meta-Analysis of the Association Between Chorioamnionitis and Severe RDS eFigure 12. Meta-Regression Plot of Association Between Chorioamnionitis and BPD36 Controlling for Difference in Gestational Age eFigure 13. Meta-Regression Plot of Association Between Chorioamnionitis and BPD36 Controlling for Risk of RDS eFigure 14. Meta-Analysis of Chorioamnionitis and BPD28, Grouped by Difference in Gestational Age eFigure 15. Meta-Analysis of Chorioamnionitis and BPD36, Grouped by Significant/Nonsignificant Difference in Gestational Age eFigure 16. Funnel Plots Assessing Publication Bias for the Association Between Chorioamnionitis and Bronchopulmonary Dysplasia eTable 1. Characteristics of All Included Studies eTable 2. Meta-Regression Analyses of Risk of BPD and Covariates eTable 3. Newcastle-Ottawa Quality Assessment of Included Studies [file jamanetwopen-2-e1914611-s001.pdf]

Supplementary Online Content

Villamor-Martinez E, Álvarez-Fuente M, Ghazi AMT, et al. Association of chorioamnionitis with bronchopulmonary dysplasia among preterm infants: a systematic review, meta-analysis, and metaregression. *JAMA Netw Open.* 2019;2(11):e1914611. doi:10.1001/jamanetworkopen.2019.14611

eFigure 1. Meta-Analysis of the Association Between Chorioamnionitis and All Bronchopulmonary Dysplasia
eFigure 2. Meta-Analysis of the Association Between Histological Chorioamnionitis and Moderate/Severe Bronchopulmonary Dysplasia
eFigure 3. Meta-Analysis of the Association Between Different Types of Chorioamnionitis and Moderate/Severe Bronchopulmonary Dysplasia
eFigure 4. Meta-Analysis of the Association Between Chorioamnionitis and Mild Bronchopulmonary Dysplasia
eFigure 5. Meta-Analysis of the Association Between Chorioamnionitis and Moderate Bronchopulmonary Dysplasia
eFigure 6. Meta-Analysis of the Association Between Chorioamnionitis and Severe Bronchopulmonary Dysplasia
eFigure 7. Meta-Analysis of the Association Between Funisitis and Bronchopulmonary Dysplasia
eFigure 8. Meta-Analysis of Chorioamnionitis and BPD28, Grouped by Use of Adjusted/Unadjusted Odds Ratios
eFigure 9. Meta-Analysis of Chorioamnionitis and BPD36, Grouped by Use of Adjusted/Unadjusted Odds Ratios
eFigure 10. Meta-Analysis of the Association Between Chorioamnionitis and all Respiratory Distress Syndrome
eFigure 11. Meta-Analysis of the Association Between Chorioamnionitis and Severe RDS
eFigure 12. Meta-Regression Plot of Association Between Chorioamnionitis and BPD36 Controlling for Difference in Gestational Age
eFigure 13. Meta-Regression Plot of Association Between Chorioamnionitis and BPD36 Controlling for Risk of RDS
eFigure 14. Meta-Analysis of Chorioamnionitis and BPD28, Grouped by Difference in Gestational Age
eFigure 15. Meta-Analysis of Chorioamnionitis and BPD36, Grouped by Significant/Nonsignificant Difference in Gestational Age
eFigure 16. Funnel Plots Assessing Publication Bias for the Association Between Chorioamnionitis and Bronchopulmonary Dysplasia
eTable 1. Characteristics of All Included Studies
eTable 2. Meta-Regression Analyses of Risk of BPD and Covariates
eTable 3. Newcastle-Ottawa Quality Assessment of Included Studies

This supplementary material has been provided by the authors to give readers additional information about their work.

© 2019 Villamor-Martinez E et al. *JAMA Network Open.*

eFigure 1. Meta-Analysis of the Association Between Chorioamnionitis and All Bronchopulmonary Dysplasia

Chorioamnionitis and BPD28 (k = 65)

Subgroup	Study name	Odds ratio	Lower limit	Upper limit	p-Value
Histological	May 2011	0.40	0.12	1.35	0.140
	Prendergast 2011	0.63	0.29	1.36	0.238
	Soraisham 2013	0.70	0.44	1.12	0.135
	Thomas 2009	0.73	0.21	2.56	0.627
	Bose 2011	0.98	0.75	1.28	0.873
	van Vliet 2012	1.23	0.43	3.54	0.700
	Dexter 2000	1.31	0.81	2.12	0.267
	Dempsey 2005	1.35	0.60	3.02	0.464
	Richardson 2006	1.50	1.02	2.20	0.038
	Kim 2008	1.52	0.57	4.05	0.406
	Shima 2013	1.52	0.95	2.44	0.081
	Sato 2011	1.57	0.99	2.50	0.056
	Bry 2015	1.71	0.09	31.92	0.718
	Ikeda 2015	1.80	1.02	3.19	0.044
	Ohyama 2002	2.24	0.98	5.10	0.056
	Ecevit 2014	2.33	0.22	24.92	0.483
	Rocha 2006	2.45	1.16	5.19	0.019
	Honma 2007	2.60	1.14	5.93	0.023
	Miyazaki 2016	2.63	2.29	3.01	0.000
	Misra 2015	2.85	0.78	10.47	0.114
	Mu 2008	2.88	1.23	6.75	0.015
	Yoon 1999	2.92	1.10	7.72	0.031
	Ghezzi 1998	2.98	0.68	13.09	0.148
	Wirbelauer 2011	3.21	1.04	9.95	0.043
	O'shea 1998	3.46	0.95	12.56	0.059
	Lee Yeri 2015	3.93	1.92	8.03	0.000
	Nishimaki 2003	3.94	1.14	13.60	0.030
	Choi 2005	4.20	1.76	10.00	0.001
	Hikino 2012	4.71	0.73	30.28	0.102
	Lee Hyun Ju 2011	4.87	1.39	17.07	0.013
	Zanardo 2002	5.27	1.08	25.78	0.040
	Choi 2006	5.63	1.99	15.88	0.001
	Watterberg 1996	6.11	1.20	31.16	0.029
	Tsiartas 2013	6.31	1.43	27.92	0.015
	Xie 2015	6.65	0.87	50.95	0.068
	Mittendorf 2005	6.85	1.45	32.35	0.015
	Xie 2016	6.86	0.75	62.96	0.089
	Zhang 2011	10.61	2.30	48.93	0.002
	Jones 2013	10.96	0.62	193.72	0.102
	De Felice 2004	13.00	2.07	81.48	0.006
	De Felice 2005	15.16	4.77	48.18	0.000
	Fukunaga 2009	23.71	1.19	474.06	0.038
	Stimac 2014	25.35	9.70	66.21	0.000
	**Histological**	**2.58**	**1.99**	**3.34**	**0.000**
Clinical	Baier 2003	0.34	0.03	3.69	0.378
	Botet 2011	0.78	0.51	1.21	0.272
	Koroglu 2013	0.94	0.17	5.34	0.948
	Wang 2014	1.02	0.17	6.02	0.980
	Barrera 2011	1.09	0.42	2.83	0.855
	Alshehri 2014	1.12	0.62	2.01	0.717
	Dexter 1999	1.23	0.72	2.10	0.452
	Schena 2015	1.48	0.86	2.55	0.161
	Bagchi 1994	1.52	0.34	6.76	0.584
	Eriksson 2014	1.59	1.45	1.75	0.000
	Gray 1997	1.81	0.63	5.26	0.273
	Baud 2000	1.92	1.33	2.77	0.001
	Lardon 2017	2.91	0.72	11.78	0.135
	Karagianni 2011	3.34	0.98	11.38	0.054
	Hyodynmaa 2012	3.41	0.66	17.50	0.142
	Stichel 2011	3.78	0.42	34.17	0.237
	Rindfleisch 1996	4.22	0.98	18.12	0.053
	Metcalfe 2017	5.61	4.79	6.58	0.000
	Karagianni 2013	9.20	0.45	186.12	0.148
	**Clinical**	**1.77**	**1.21**	**2.61**	**0.004**
Microbiological	Miralles 2008	1.16	0.24	5.53	0.856
	Abele-Horn 1997	3.94	1.23	12.62	0.021
	Abele-Horn 1998	4.73	1.32	16.92	0.017
	**Microbiological**	**2.99**	**1.03**	**8.68**	**0.044**
Overall	**OVERALL**	**2.32**	**1.88**	**2.86**	**0.000**

Heterogeneity: $p < 0.001$; $I^2 = 84\%$

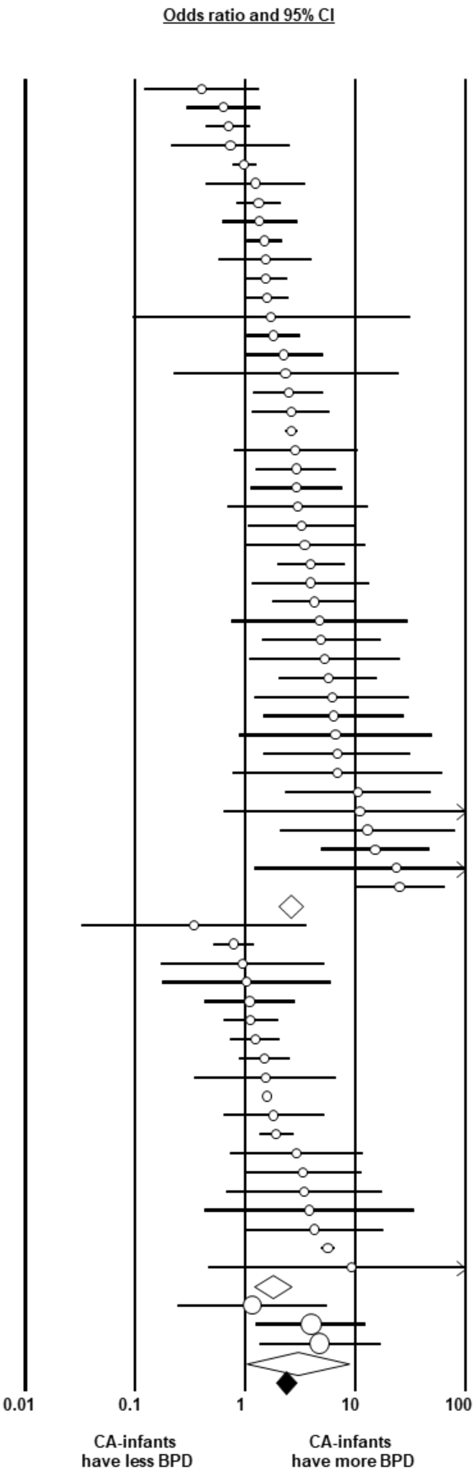

BPD: bronchopulmonary dysplasia; BPD28: bronchopulmonary dysplasia defined as supplemental oxygen requirement on postnatal day 28; CA: chorioamnionitis; CI: confidence interval; k: number of studies.

© 2019 Villamor-Martinez E et al. *JAMA Network Open.*

eFigure 2. Meta-Analysis of the Association Between Histological Chorioamnionitis and Moderate/Severe Bronchopulmonary Dysplasia

Histological chorioamnionitis and BPD36 (k = 68)

Study name	Odds ratio	Lower limit	Upper limit	p-Value
Durrmeyer 2012	0.29	0.13	0.65	0.003
Oh 2015	0.38	0.13	1.13	0.082
Young 2005	0.43	0.23	0.81	0.010
Kaukola 2009	0.50	0.21	1.21	0.124
Leroy 2018	0.56	0.18	1.73	0.315
Thomas 2009	0.57	0.10	3.22	0.520
Plakkal 2013	0.58	0.38	0.89	0.012
May 2011	0.65	0.19	2.21	0.491
Van Marter 2002	0.65	0.40	1.07	0.092
Kewitz 2008	0.70	0.32	1.54	0.373
McGowan (h) 2009	0.78	0.35	1.75	0.550
Bordigato 2011	0.80	0.16	3.88	0.782
Nasef (h) 2013	0.81	0.44	1.48	0.489
Kandasamy (h) 2015	0.83	0.41	1.68	0.597
Prendergast 2011	0.85	0.39	1.81	0.668
Watterberg 1999	0.90	0.25	3.25	0.870
Pappas (h) 2014	0.92	0.77	1.11	0.391
Mailaparambil 2010	0.93	0.46	1.85	0.827
Nicaise 2002	0.93	0.23	3.83	0.923
Trevisanuto 2013	0.94	0.36	2.47	0.896
Kim 2008	0.97	0.38	2.50	0.948
Ahn 2012	0.98	0.55	1.76	0.952
Gantar 2011 (h)	1.03	0.42	2.51	0.953
Kent 2004	1.04	0.59	1.83	0.897
Redline (h) 2002	1.05	0.67	1.64	0.824
Kalra 2014	1.07	0.38	3.01	0.895
Suppiej 2009	1.08	0.46	2.52	0.867
Fujioka 2014	1.08	0.45	2.63	0.860
Arayici 2014	1.10	0.49	2.48	0.810
Gantar 2011 (c)	1.11	0.39	3.14	0.851
Strunk 2012	1.11	0.82	1.48	0.505
Streubel 2008	1.12	0.52	2.40	0.768
Torchin 2017	1.14	0.82	1.59	0.424
Been 2010	1.23	0.65	2.33	0.529
Soliman 2017	1.33	0.79	2.24	0.279
Smit 2015	1.41	0.73	2.71	0.301
Kim 2015	1.50	0.83	2.72	0.178
Hendson 2011	1.55	1.07	2.23	0.019
Paananen 2009	1.55	0.69	3.50	0.292
Liu 2014	1.60	0.58	4.36	0.362
Lee Hyun Ju 2011	1.61	0.87	2.98	0.129
Stepan 2016	1.69	0.08	36.08	0.739
Seliga-Sizecka 2013	1.75	1.00	3.08	0.051
Lau 2005	1.88	1.32	2.66	0.000
Kim 2004	1.91	1.04	3.49	0.036
McGowan (c) 2009	1.94	0.64	5.84	0.240
Shima 2013	1.94	1.06	3.53	0.030
Rocha 2011	1.99	0.85	4.65	0.113
Lohmann 2014	2.00	0.32	12.33	0.455
Ogunyemi 2003	2.03	1.40	2.94	0.000
Zanardo 2008	2.19	1.16	4.13	0.015
Misra 2015	2.25	0.36	13.97	0.384
Polam 2005	2.38	1.26	4.49	0.008
Ikeda 2015	2.43	1.13	5.21	0.023
Rocha 2010	2.44	1.25	4.79	0.009
Watterberg 2004	2.54	1.25	5.19	0.010
Bry 2015	2.67	0.43	16.39	0.290
Dessardo 2014	2.87	1.60	5.16	0.000
Mestan 2010	3.04	1.26	7.35	0.013
Shima 2011	3.08	1.29	7.37	0.012
Mehta 2006	3.17	1.38	7.26	0.007
Curley 2003	3.24	1.09	9.58	0.034
Choi 2008	3.81	1.27	11.39	0.017
Perrone 2012	3.82	1.54	9.44	0.004
Viscardi 2004	3.91	1.46	10.44	0.007
Natarajan 2008	3.96	0.93	16.90	0.063
Chisholm 2016	6.08	1.54	23.95	0.010
Erdemir 2013	21.50	3.50	131.90	0.001
**OVERALL**	**1.34**	**1.17**	**1.54**	**0.000**

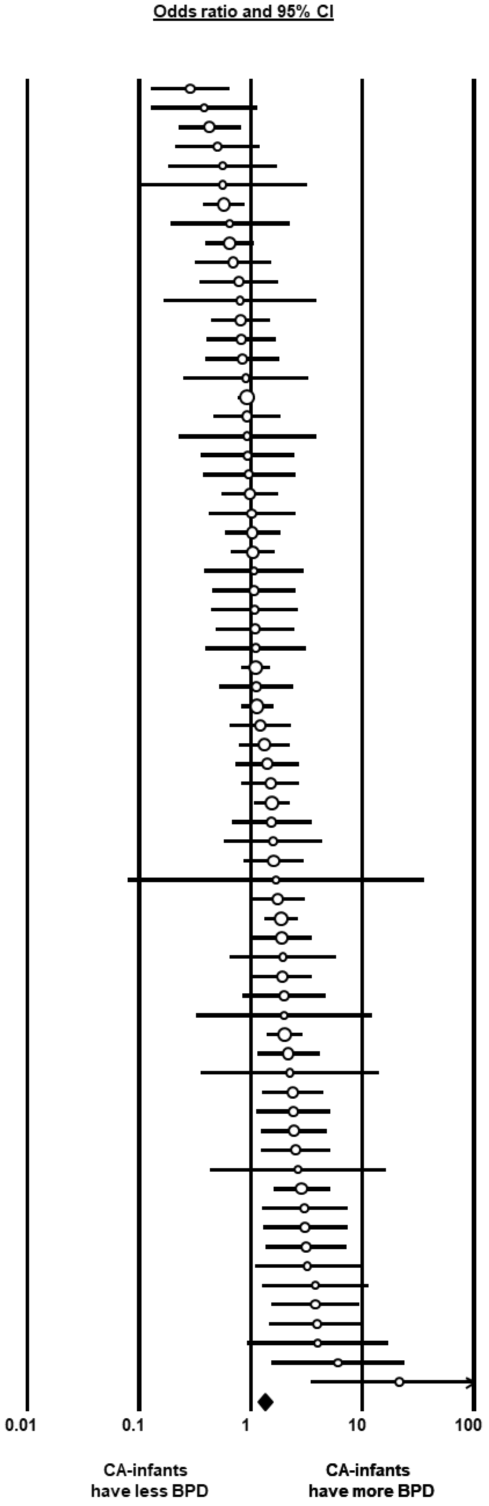

Heterogeneity: p = <0.001; I^2 = 62%

BPD: bronchopulmonary dysplasia; BPD36: bronchopulmonary dysplasia defined as supplemental oxygen requirement at the postmenstrual age of 36 weeks; CA: chorioamnionitis; CI: confidence interval; k: number of studies

© 2019 Villamor-Martinez E et al. *JAMA Network Open.*

eFigure 3. Meta-Analysis of the Association Between Different Types of Chorioamnionitis and Moderate/Severe Bronchopulmonary Dysplasia

Clinical. histological + clinical and microbiological chorioamnionitis and BPD36 (k = 40)

Subgroup	Study name	Odds ratio	Lower limit	Upper limit	p-Value
Clinical	Cederqvist 2003	0.27	0.05	1.36	0.112
	Guimaraes 2010	0.34	0.10	1.15	0.083
	Baker 2012	0.37	0.04	3.23	0.368
	Nasef (c) 2013	0.39	0.13	1.19	0.098
	Kandasamy (c) 2015	0.44	0.12	1.62	0.214
	Botet 2011	0.62	0.33	1.17	0.139
	Colaizy 2007	0.63	0.25	1.62	0.341
	Serenius 2004	0.64	0.27	1.51	0.308
	Mahlman 2017	0.67	0.33	1.35	0.258
	Kazzi 2004	0.69	0.29	1.66	0.408
	Lapcharoensap 2015	1.01	0.90	1.13	0.875
	Tokuriki 2015	1.03	0.20	5.33	0.973
	Lodha 2014	1.05	0.78	1.42	0.746
	Brener Dick 2017	1.11	0.39	3.15	0.852
	Morrow 2017	1.18	0.78	1.80	0.433
	Redline (c) 2002	1.28	0.65	2.49	0.476
	Soraisham 2009	1.39	1.11	1.74	0.004
	Stichel 2011	1.40	0.43	4.51	0.573
	Gagliardi 2014	1.41	1.21	1.64	0.000
	Klinger 2013	1.51	1.25	1.84	0.000
	Gonzáles-Luis 2002	1.63	0.63	4.20	0.317
	Sampath 2015	1.63	0.96	2.77	0.071
	Garcia-Munoz Rodrigo 2014	1.72	1.50	1.98	0.000
	Demiral 2009	1.96	0.62	6.18	0.252
	Hansen 2010	1.98	0.72	5.44	0.182
	Lin 2005	2.12	0.77	5.86	0.148
	Novitsky 2015	2.41	1.42	4.08	0.001
	Lardon-Fernandez 2017	3.11	0.81	12.00	0.099
	Li 2015	5.14	1.45	18.22	0.011
	Ameenudeen 2007	6.29	2.11	18.72	0.001
	Jonsson 1998	32.00	1.23	832.52	0.037
	**Clinical**	**1.24**	**1.03**	**1.49**	**0.020**
Histological + clinical	Schlapbach 2010	0.50	0.13	1.93	0.315
	Pappas (Both) 2014	1.04	0.83	1.29	0.759
	**Histological + clin**	**0.91**	**0.48**	**1.72**	**0.769**
Microbiological	Miralles 2008	0.29	0.03	2.98	0.299
	Khan 2006	0.80	0.42	1.52	0.486
	Lamboley-Gilmert 2003	1.17	0.80	1.70	0.424
	Rojas 2012	1.34	0.38	4.76	0.648
	Fung 2003	1.56	0.46	5.31	0.481
	Hitti 2001	2.28	0.72	7.22	0.161
	Kirchner 2007	4.33	0.87	21.60	0.074
	**Microbiological**	**1.25**	**0.80**	**1.97**	**0.330**

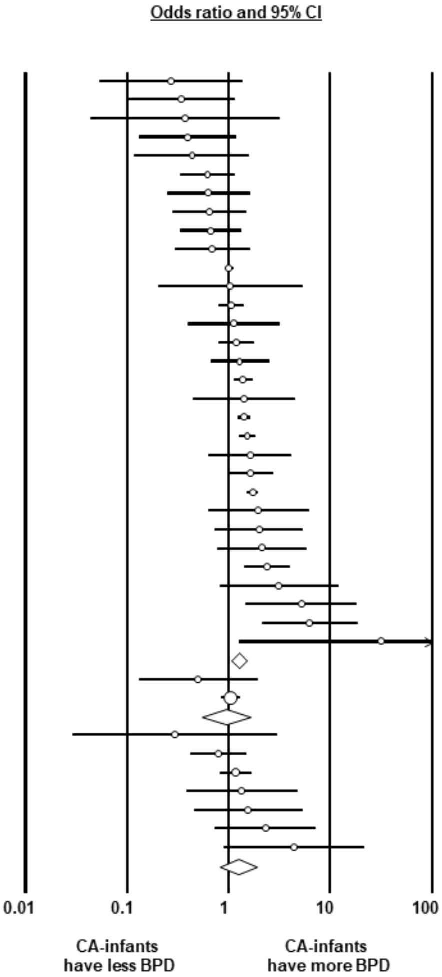

BPD: bronchopulmonary dysplasia; BPD36: bronchopulmonary dysplasia defined as supplemental oxygen requirement at the postmenstrual age of 36 weeks; CA: chorioamnionitis; CI: confidence interval; k: number of studies

© 2019 Villamor-Martinez E et al. *JAMA Network Open.*

eFigure 4. Meta-Analysis of the Association Between Chorioamnionitis and Mild Bronchopulmonary Dysplasia

Chorioamnionitis and mild BPD (k = 15)

Subgroup	Study name	Odds ratio	Lower limit	Upper limit	p-Value
Histological	May 2011	0.38	0.07	2.21	0.280
	Bry 2015	0.44	0.07	2.74	0.382
	Lee Hyun Ju 2011	0.61	0.31	1.21	0.157
	Prendergast 2011	0.63	0.23	1.73	0.366
	Thomas 2009	0.98	0.25	3.76	0.974
	Ikeda 2015	0.98	0.53	1.82	0.948
	Shima 2013	1.02	0.60	1.75	0.930
	Kim 2008	1.58	0.59	4.27	0.364
	Misra 2015	2.15	0.52	9.00	0.293
	Streubel 2008	2.37	1.16	4.86	0.018
	**Histological**	1.01	0.62	1.63	0.982
Clinical	Botet 2011	0.98	0.61	1.58	0.943
	Lardon-Fernandez 2017	1.17	0.28	4.78	0.832
	Stichel 2011	1.20	0.38	3.79	0.756
	Baud 2000	4.21	2.90	6.11	0.000
	**Clinical**	1.72	0.85	3.47	0.131
Microbiological	Miralles 2008	5.10	0.47	55.89	0.182
	**OVERALL**	1.23	0.83	1.83	0.296

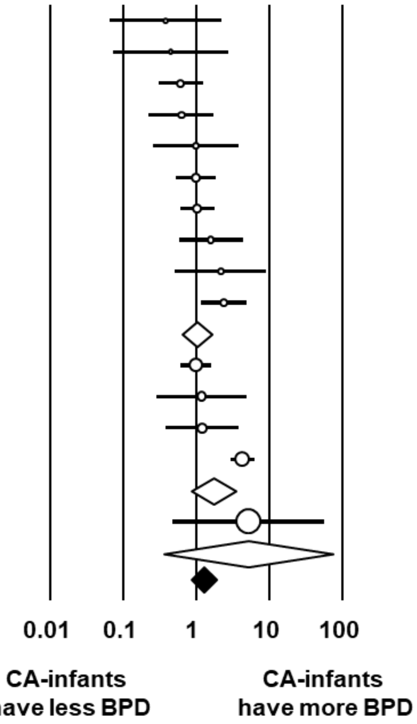

Heterogeneity: $p < 0.001$; $I^2 = 74\%$

BPD: bronchopulmonary dysplasia; CA: chorioamnionitis; CI: confidence interval; k: number of studies.

© 2019 Villamor-Martinez E et al. *JAMA Network Open.*

eFigure 5. Meta-Analysis of the Association Between Chorioamnionitis and Moderate Bronchopulmonary Dysplasia

Chorioamnionitis and moderate BPD (k = 7)

Subgroups	Study name	Odds ratio	Lower limit	Upper limit	p-Value
Histological	May 2011	0.47	0.10	2.19	0.338
	Thomas 2009	0.57	0.10	3.22	0.520
	Prendergast 2011	0.67	0.29	1.53	0.341
	Ikeda 2015	1.97	0.74	5.28	0.175
	Misra 2015	2.25	0.36	13.97	0.384
	**Histological**	**0.96**	**0.55**	**1.67**	**0.889**
Clinical	Lardon-Fernandez 2017	0.77	0.09	6.52	0.811
	Sampath 2015	1.63	0.92	2.90	0.097
	**Clinical**	**1.53**	**0.83**	**2.84**	**0.176**
	**OVERALL**	**1.18**	**0.78**	**1.79**	**0.425**

Heterogeneity: $p = 0.346$; $I^2 = 11\%$

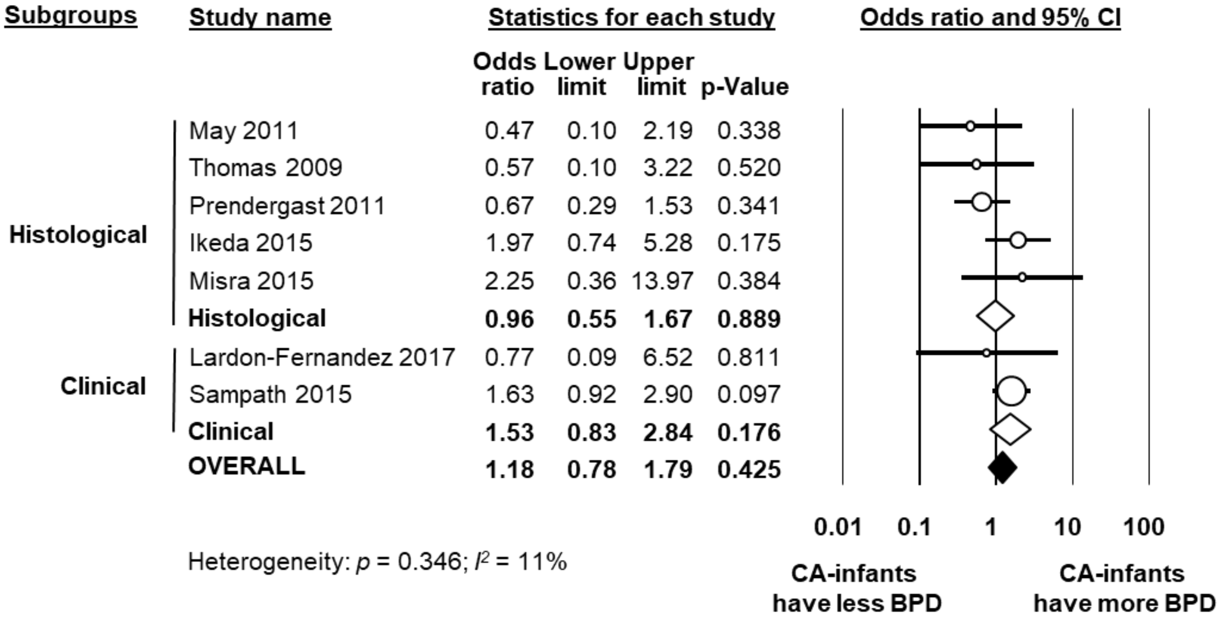

BPD: bronchopulmonary dysplasia; CI: confidence interval; k: number of studies.

© 2019 Villamor-Martinez E et al. *JAMA Network Open.*

eFigure 6. Meta-Analysis of the Association Between Chorioamnionitis and Severe Bronchopulmonary Dysplasia

Chorioamnionitis and severe BPD (k = 8)

Subgroups	Study name	Odds ratio	Lower limit	Upper limit	p-Value
Histological	Bose 2011	0.71	0.44	1.16	0.171
	May 2011	1.12	0.24	5.17	0.887
	Prendergast 2011	1.52	0.49	4.73	0.468
	Ikeda 2015	2.43	0.83	7.14	0.107
	**Histological**	**1.15**	**0.62**	**2.16**	**0.658**
Clinical	Guo 2015	0.98	0.35	2.75	0.966
	EXPRESS group 2010	1.00	0.60	1.67	1.000
	Sampath 2015	1.17	0.63	2.20	0.617
	Lardon-Fernandez 2017	8.07	1.66	39.27	0.010
	**Clinical**	**1.31**	**0.73**	**2.34**	**0.360**
	**OVERALL**	**1.24**	**0.81**	**1.89**	**0.330**

Heterogeneity: $p = 0.110$; $I^2 = 40\%$

Odds ratio and 95% CI

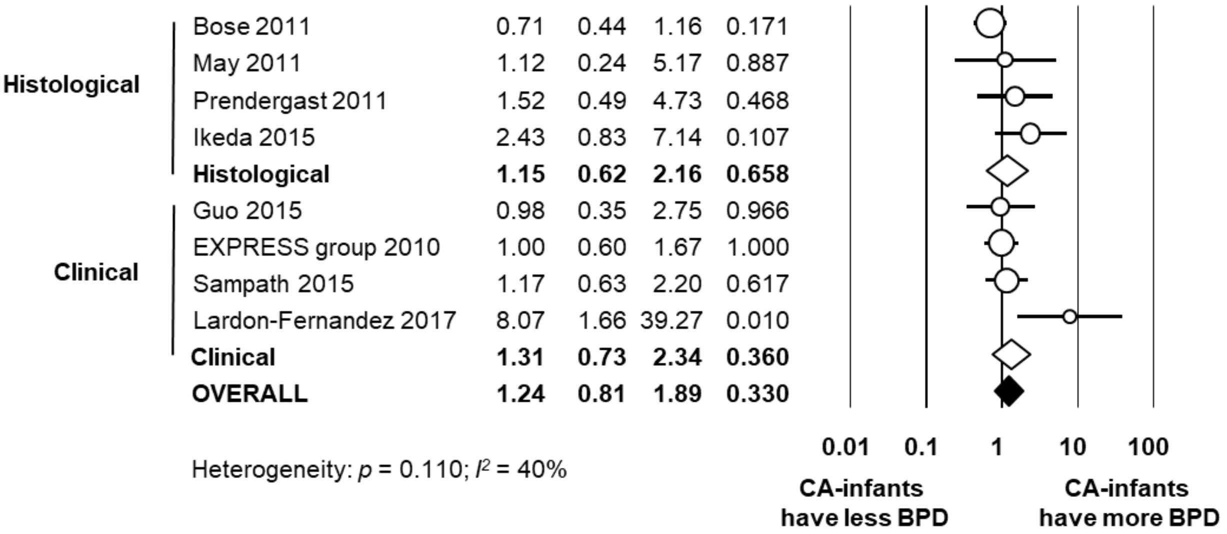

BPD: bronchopulmonary dysplasia; CI: confidence interval; k: number of studies.

© 2019 Villamor-Martinez E et al. *JAMA Network Open.*

eFigure 7. Meta-Analysis of the Association Between Funisitis and Bronchopulmonary Dysplasia

Funisitis vs. chorioamnionitis without funisitis and BPD (k = 20)

Subgroup	Study name	Odds ratio	Lower limit	Upper limit	p-Value
BPD28	Ohyama 2002	0.76	0.14	4.03	0.747
	Richardson 2006	0.79	0.45	1.36	0.388
	Kim 2015	0.90	0.35	2.33	0.835
	Lee Yeri 2015	1.42	0.55	3.68	0.474
	Tsiartas 2013	4.16	1.49	11.61	0.006
	**BPD28**	**1.26**	**0.61**	**2.59**	**0.530**
BPD36	Liu 2014	0.41	0.10	1.69	0.217
	Kim 2008	0.43	0.09	2.03	0.286
	Iwatani 2013	0.45	0.14	1.45	0.184
	Gantar 2011	0.80	0.17	3.66	0.774
	Mestan 2010	0.86	0.33	2.24	0.753
	Torchin 2017	0.86	0.50	1.50	0.598
	Kent 2004	0.89	0.35	2.26	0.813
	Been 2010	0.94	0.36	2.48	0.906
	Smit 2015	1.01	0.40	2.55	0.986
	Plakkal 2013	1.16	0.63	2.13	0.630
	Lau 2005	1.21	0.69	2.11	0.508
	Trevisanuto 2013	1.23	0.44	3.46	0.697
	Rocha 2011	2.92	0.77	11.12	0.117
	Thomas 2009	3.33	0.33	34.12	0.310
	Dessardo 2014	38.50	11.47	129.18	0.000
	**BPD36**	**1.19**	**0.77**	**1.83**	**0.429**

Heterogeneity: $p = 0.110$; $I^2 = 40\%$

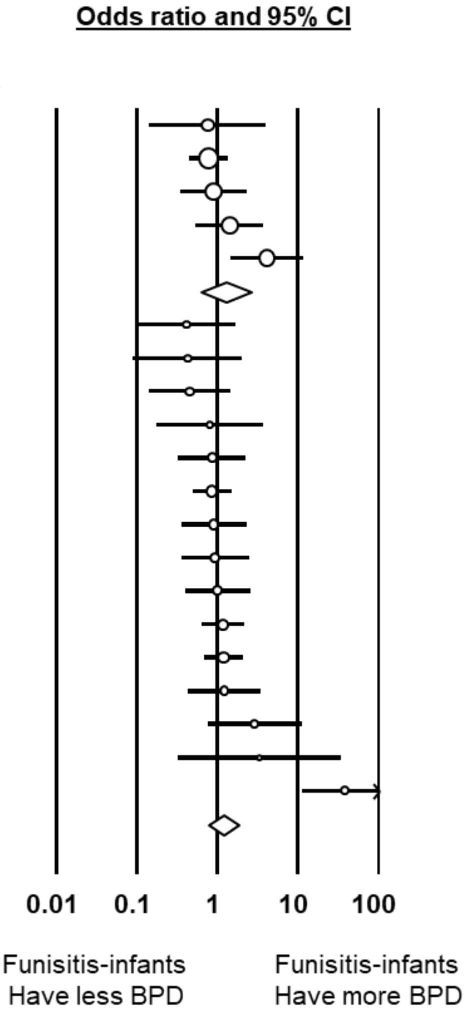

BPD: bronchopulmonary dysplasia; BPD28: bronchopulmonary dysplasia defined as supplemental oxygen requirement on postnatal day 28; BPD36: bronchopulmonary dysplasia defined as supplemental oxygen requirement at the postmenstrual age of 36 weeks; CA: chorioamnionitis; CI: confidence interval; k: number of studies

© 2019 Villamor-Martinez E et al. *JAMA Network Open.*

eFigure 8. Meta-Analysis of Chorioamnionitis and BPD28, Grouped by Use of Adjusted/Unadjusted Odds Ratios

Subgroup	Study name	Odds ratio	Lower limit	Upper limit	p-Value
Adjusted	Soraisham 2009	0.85	0.58	1.24	0.402
	Baud 2000	0.96	0.36	2.57	0.935
	Eriksson 2014	1.33	1.19	1.48	0.000
	Miyazaki 2016	1.68	1.41	2.01	0.000
	Rocha 2006	2.66	0.36	19.63	0.337
	Choi 2008	2.80	0.29	27.05	0.374
	Mu 2008	3.02	1.23	7.38	0.015
	Viscardi 2004	3.13	1.20	8.17	0.020
	Ohyama 2002	3.20	1.40	7.31	0.006
	Jonsson 1998	3.70	1.09	12.55	0.036
	Abele-Horn 1998	6.18	1.04	36.64	0.045
	**Adjusted overall**	1.68	1.28	2.21	0.000
Unadjusted	Soraisham 2009	1.39	1.11	1.74	0.004
	Eriksson 2014	1.59	1.45	1.75	0.000
	Baud 2000	1.92	1.33	2.77	0.001
	Ohyama 2002	2.24	0.98	5.10	0.056
	Rocha 2006	2.45	1.16	5.19	0.019
	Miyazaki 2016	2.63	2.29	3.01	0.000
	Mu 2008	2.88	1.23	6.75	0.015
	Jonsson 1998	3.20	1.23	8.32	0.017
	Choi 2008	3.81	1.27	11.39	0.017
	Viscardi 2004	3.91	1.46	10.44	0.006
	Abele-Horn 1998	3.94	1.23	12.62	0.021
	**Unadjusted overall**	2.17	1.71	2.76	0.000

Heterogeneity: $p < 0.001$; $I^2 = 78\%$

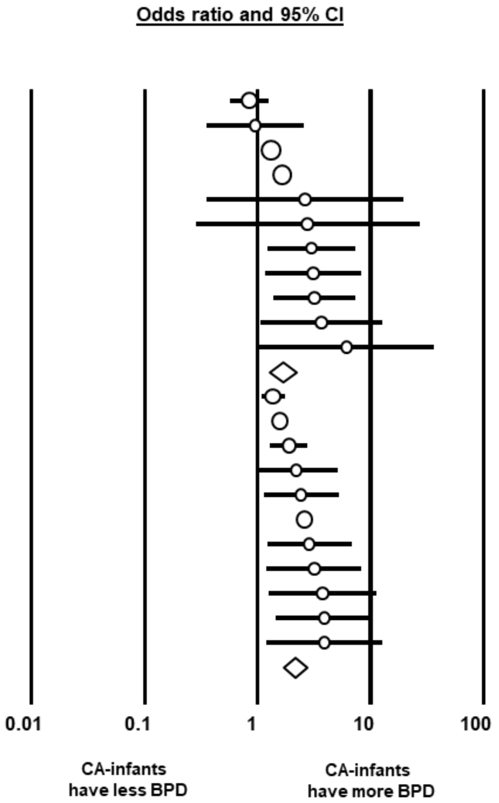

BPD: bronchopulmonary dysplasia; BPD28: bronchopulmonary dysplasia defined as supplemental oxygen requirement on postnatal day 28; CA: chorioamnionitis; CI: confidence interval.

© 2019 Villamor-Martinez E et al. *JAMA Network Open.*

eFigure 9. Meta-Analysis of Chorioamnionitis and BPD36, Grouped by Use of Adjusted/Unadjusted Odds Ratios

Subgroup	Study name	Odds ratio	Lower limit	Upper limit	p-Value
Adjusted	Ahn 2012	0.18	0.05	0.64	0.008
	Van Marter 2002	0.60	0.33	1.09	0.091
	Redline (h) 2002	0.70	0.40	1.23	0.212
	Soraisham 2009	0.85	0.58	1.24	0.402
	Kim 2015	0.85	0.40	1.80	0.672
	Plakkal 2013	0.93	0.86	1.00	0.059
	Garcia-Munoz Rodrigo 2014	0.95	0.75	1.21	0.671
	Baud 2000	0.96	0.36	2.57	0.935
	KENT 2004	1.16	0.53	2.55	0.712
	Paananen 2009	1.20	0.40	3.60	0.745
	Eriksson 2014	1.33	1.19	1.48	0.000
	Miyazaki 2016	1.68	1.41	2.01	0.000
	Rocha 2010	1.97	0.81	4.78	0.137
	Rocha 2006	2.66	0.36	19.63	0.337
	Choi 2008	2.80	0.29	27.05	0.374
	Mu 2008	3.02	1.23	7.38	0.015
	Viscardi 2004	3.13	1.20	8.17	0.020
	Ohyama 2002	3.20	1.40	7.31	0.006
	Jonsson 1998	3.70	1.09	12.55	0.036
	Abele-Horn 1998	6.18	1.04	36.64	0.045
	Ameenudeen 2007	9.00	1.60	50.71	0.013
	**Adjusted overall**	**1.25**	**1.01**	**1.54**	**0.039**
Unadjusted	Plakkal 2013	0.58	0.38	0.89	0.012
	Van Marter 2002	0.65	0.40	1.07	0.092
	Ahn 2012	0.98	0.55	1.76	0.952
	KENT 2004	1.04	0.59	1.83	0.897
	Redline (h) 2002	1.05	0.67	1.64	0.824
	Soraisham 2009	1.39	1.11	1.74	0.004
	Kim 2015	1.50	0.83	2.72	0.178
	Paananen 2009	1.55	0.69	3.50	0.292
	Eriksson 2014	1.59	1.45	1.75	0.000
	Garcia-Munoz Rodrigo 2014	1.72	1.50	1.98	0.000
	Baud 2000	1.85	1.01	3.40	0.048
	Ohyama 2002	2.24	0.98	5.10	0.056
	Rocha 2010	2.44	1.25	4.79	0.009
	Rocha 2006	2.45	1.16	5.19	0.019
	Miyazaki 2016	2.63	2.29	3.01	0.000
	Mu 2008	2.88	1.23	6.75	0.015
	Jonsson 1998	3.20	1.23	8.32	0.017
	Choi 2008	3.81	1.27	11.39	0.017
	Viscardi 2004	3.91	1.46	10.44	0.006
	Abele-Horn 1998	3.94	1.23	12.62	0.021
	Ameenudeen 2007	6.29	2.11	18.72	0.001
	**Unadjusted overall**	**1.65**	**1.37**	**2.00**	**0.000**

Heterogeneity: $p < 0.001$; $I^2 = 87\%$

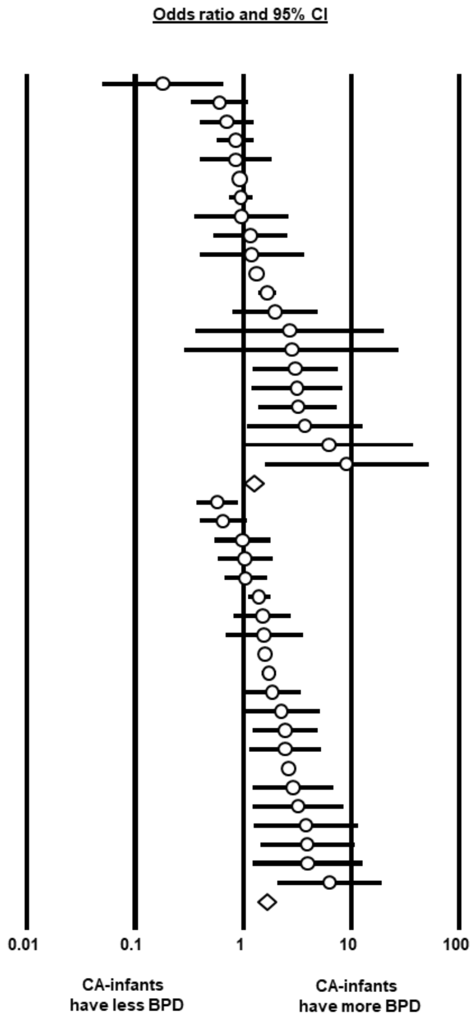

BPD: bronchopulmonary dysplasia; BPD36: bronchopulmonary dysplasia, defined as supplemental oxygen requirement at the postmenstrual age of 36 weeks; CA: chorioamnionitis; CI: confidence interval.

© 2019 Villamor-Martinez E et al. *JAMA Network Open.*

eFigure 10. Meta-Analysis of the Association Between Chorioamnionitis and all Respiratory Distress Syndrome

Chorioamnionitis and respiratory distress syndrome (k = 48)

Subgroup	Study name	Odds ratio	Lower limit	Upper limit	p-Value
	Watterberg 1996	0.11	0.02	0.63	0.013
	Liu 2014	0.16	0.05	0.49	0.001
	Ohyama 2002	0.17	0.08	0.38	0.000
	Choi 2008	0.27	0.09	0.80	0.019
	Dempsey 2005	0.48	0.30	0.75	0.001
	Thomas 2009	0.62	0.17	2.30	0.475
	Young 2005	0.63	0.32	1.24	0.180
	Arayici 2014	0.64	0.40	1.03	0.067
	Smit2015	0.67	0.41	1.07	0.096
	Plakkal 2013	0.73	0.37	1.45	0.366
	Soraisham 2013	0.73	0.39	1.39	0.343
	Prendergast 2011	0.74	0.33	1.68	0.476
	Ahn 2012	0.89	0.53	1.49	0.653
	Miyazaki 2016	0.89	0.78	1.02	0.104
	Richardson 2006	0.92	0.67	1.25	0.585
	Lau 2005	0.93	0.64	1.34	0.704
Histological	Xie 2015	0.94	0.57	1.55	0.808
	Been 2010	0.98	0.62	1.56	0.936
	Misra 2015	1.00	0.06	17.18	1.000
	Kent 2004	1.02	0.56	1.88	0.940
	Kim 2015	1.04	0.63	1.73	0.878
	Stepan 2016	1.25	0.55	2.87	0.595
	Dessardo 2014	1.30	0.75	2.24	0.350
	Soraisham 2009	1.30	1.06	1.61	0.013
	Zanardo. 2008	1.31	0.73	2.35	0.359
	Rocha 2006	1.47	0.96	2.25	0.073
	Seliga-Sizecka 2013	1.75	1.15	2.66	0.009
	Lee Hyun Ju 2011	1.91	0.66	5.57	0.234
	Ecevit 2014	2.00	0.21	18.69	0.543
	Hendson 2011	2.02	0.97	4.22	0.061
	De Felice 2005	2.08	0.97	4.48	0.062
	Bordigato 2011	2.18	0.33	14.36	0.417
	Tsiartas 2013	2.28	1.23	4.24	0.009
	Mu 2008	2.63	1.02	6.78	0.046
	Lee Yeri 2015	2.83	1.78	4.50	0.000
	Chisholm 2016	5.71	1.28	25.55	0.023
	Erdemir 2013	12.00	2.69	53.62	0.001
	Jones 2013	17.76	1.02	307.69	0.048
	**Histological**	1.04	0.84	1.27	0.737
	Baud 2000	0.56	0.39	0.79	0.001
	Gagliardi 2014	0.77	0.62	0.95	0.014
	Botet 2011	0.78	0.50	1.23	0.286
Clinical	Dexter 1999	1.13	0.47	2.70	0.788
	Gray 1997	1.21	0.42	3.54	0.724
	Garcia-Munoz Rodrigo 2014	1.47	1.30	1.66	0.000
	Metcalfe 2017	2.63	2.40	2.88	0.000
	**Clinical**	1.09	0.72	1.67	0.676
Microbiological	Hitti 2001	3.57	1.37	9.27	0.009
	Abele-Horn 1997	9.98	4.16	23.95	0.000
	**Microbiological**	6.09	2.32	15.96	0.000
	Fung 2003	1.12	0.21	5.97	0.898
Clin + histol	**Both**	1.12	0.16	7.91	0.912
	**OVERALL**	1.12	0.93	1.34	0.240

Heterogeneity: $p < 0.001$; $I^2 = 90\%$

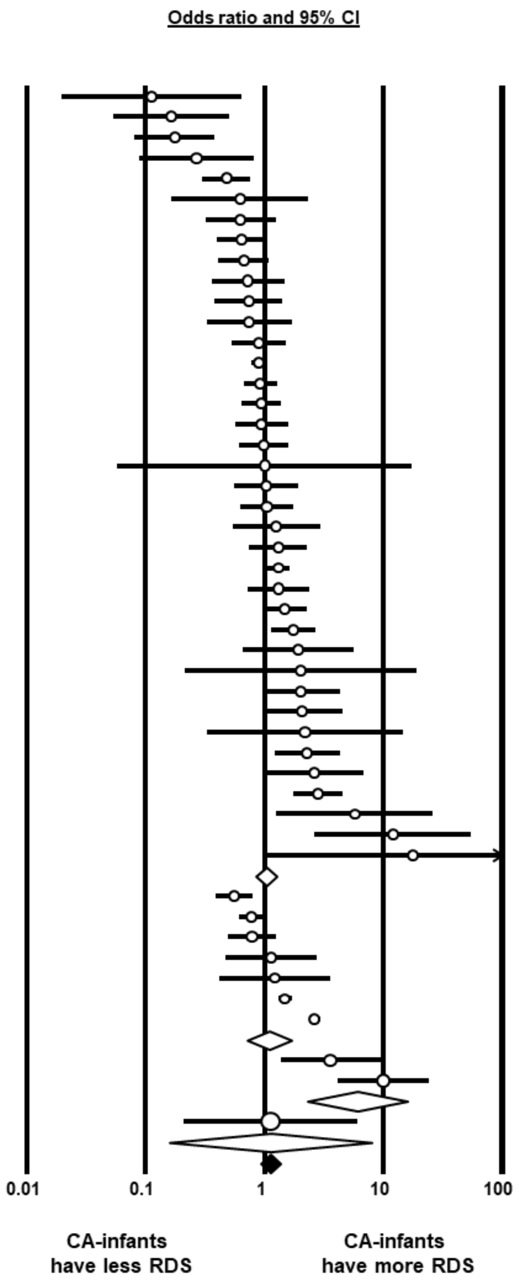

CA: chorioamnionitis; CI: confidence interval; k: number of studies; RDS: respiratory distress syndrome.

© 2019 Villamor-Martinez E et al. *JAMA Network Open.*

eFigure 11. Meta-Analysis of the Association Between Chorioamnionitis and Severe RDS

Chorioamnionitis and severe respiratory distress syndrome (k = 20)

Subgroup	Study name	Odds ratio	Lower limit	Upper limit	p-Value
Histological	Ohyama 2002	0.11	0.05	0.25	0.000
Histological	Liu 2014	0.16	0.05	0.49	0.001
Histological	Thomas 2009	0.62	0.17	2.30	0.475
Histological	Soraisham 2013	0.62	0.40	0.98	0.040
Histological	Arayici 2014	0.64	0.40	1.03	0.067
Histological	Plakkal 2013	0.69	0.47	1.02	0.062
Histological	Prendergast 2011	0.74	0.33	1.68	0.476
Histological	Smit 2015	0.78	0.55	1.09	0.144
Histological	Been 2010	0.96	0.61	1.53	0.871
Histological	Misra 2015	1.00	0.06	17.18	1.000
Histological	Stepan 2016	1.25	0.55	2.87	0.595
Histological	Dessardo 2014	1.30	0.75	2.24	0.350
Histological	Soraisham 2009	1.30	1.06	1.61	0.013
Histological	Tsiartas 2013	2.28	1.23	4.24	0.009
Histological	Lau 2005	2.44	1.87	3.19	0.000
Histological	Mu 2008	2.63	1.02	6.78	0.046
Histological	Lee Yeri 2015	2.83	1.78	4.50	0.000
Histological	Jones 2013	17.76	1.02	307.69	0.048
Histological	**Histological**	**0.99**	**0.74**	**1.32**	**0.933**
Clinical	Gagliardi 2014	1.40	1.21	1.61	0.000
Clinical	Metcalfe 2017	2.08	1.86	2.33	0.000
Clinical	**Clinical**	**1.71**	**0.84**	**3.45**	**0.137**
	**OVERALL**	**1.07**	**0.82**	**1.39**	**0.628**

Heterogeneity: $p < 0.001$; $I^2 = 90\%$

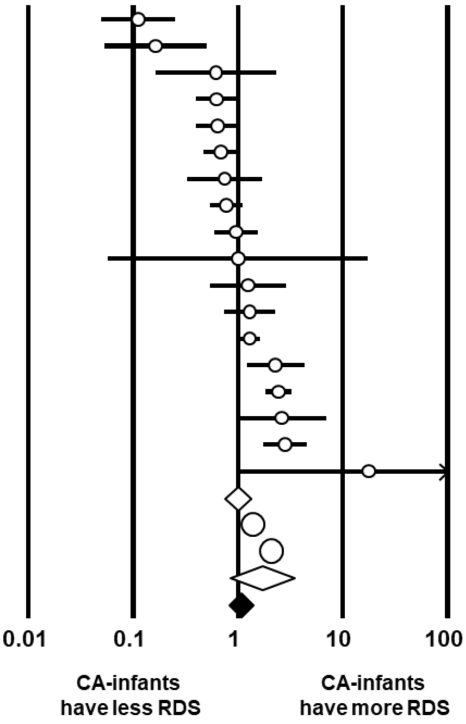

CI: confidence interval; k: number of studies; Severe RDS: respiratory distress syndrome, defined as necessity of surfactant and/or mechanical ventilation.

© 2019 Villamor-Martinez E et al. *JAMA Network Open.*

eFigure 12. Meta-Regression Plot of Association Between Chorioamnionitis and BPD36 Controlling for Difference in Gestational Age

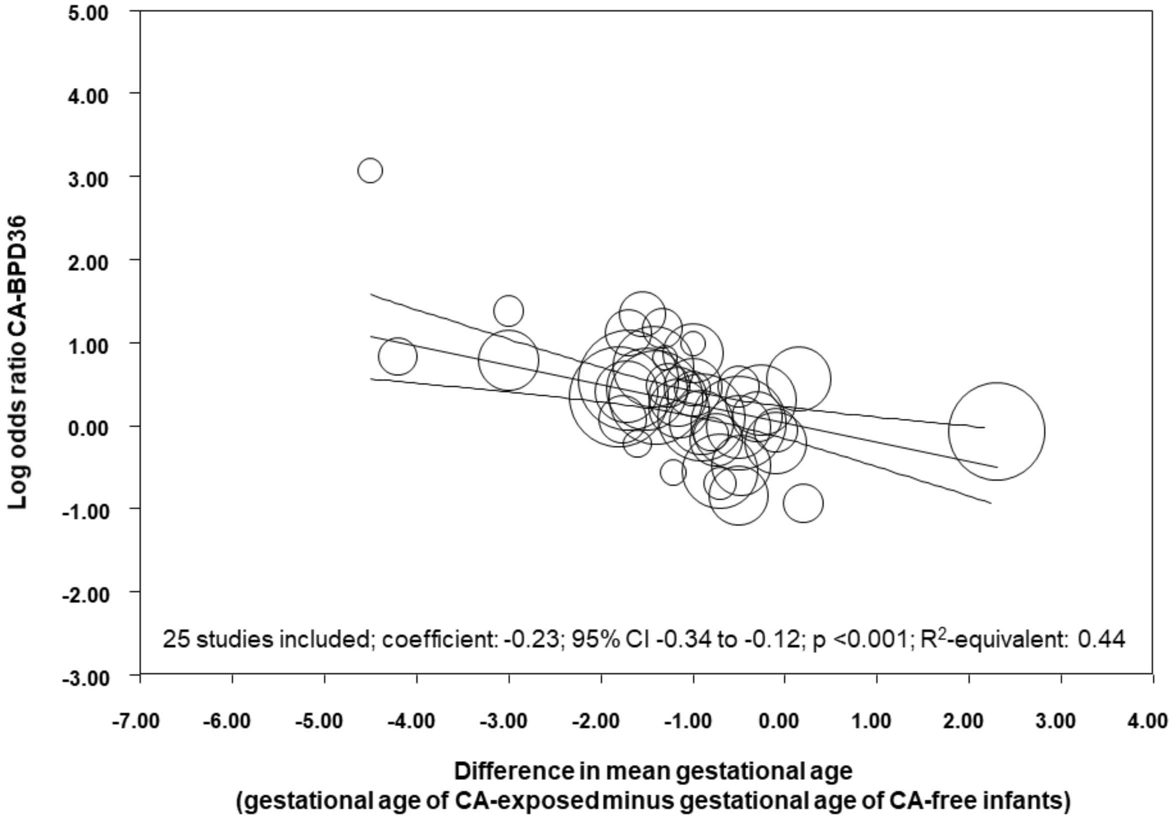

BPD36: bronchopulmonary dysplasia, defined as supplemental oxygen requirement at the postmenstrual age of 36 weeks; CA: chorioamnionitis; CI: confidence interval.

© 2019 Villamor-Martinez E et al. *JAMA Network Open.*

eFigure 13. Meta-Regression Plot of Association Between Chorioamnionitis and BPD36 Controlling for Risk of RDS

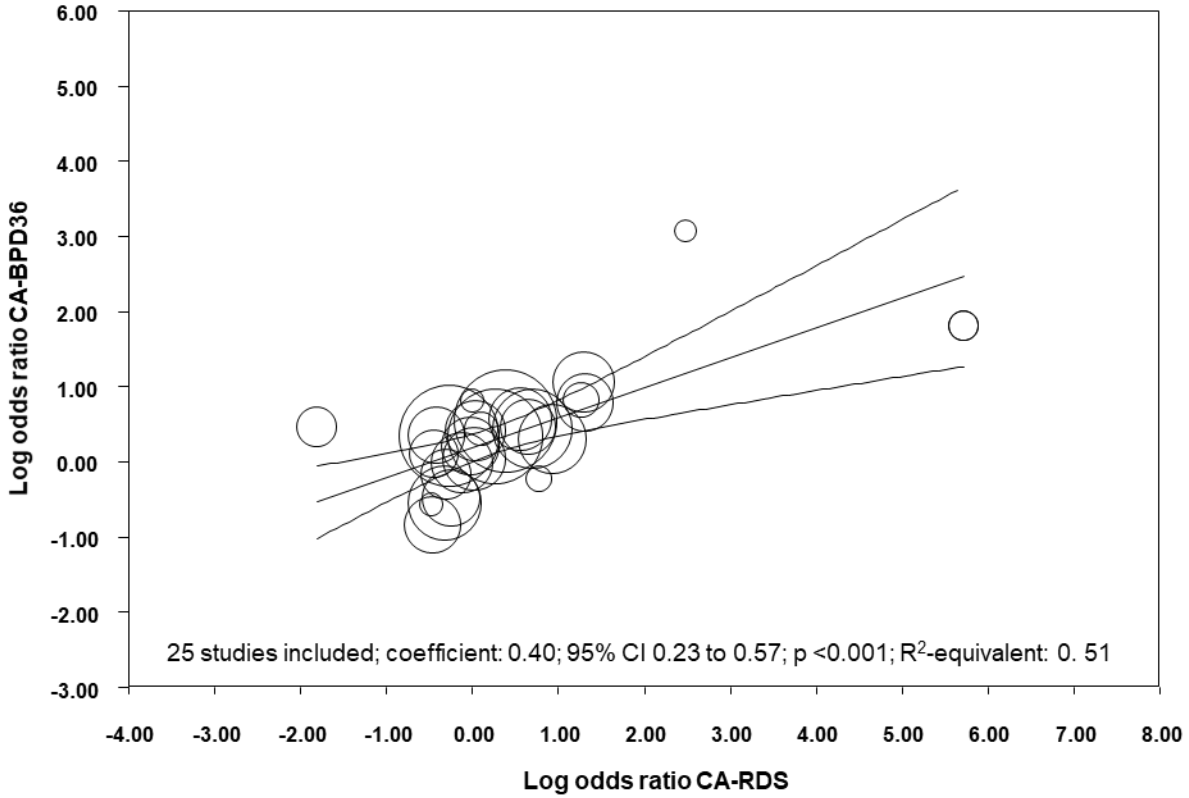

BPD36: bronchopulmonary dysplasia, defined as supplemental oxygen requirement at the postmenstrual age of 36 weeks; CI: confidence interval; RDS: respiratory distress syndrome.

© 2019 Villamor-Martinez E et al. *JAMA Network Open.*

eFigure 14. Meta-Analysis of Chorioamnionitis and BPD28, Grouped by Difference in Gestational Age

Subgroup	Study name	Odds ratio	Lower limit	Upper limit	p-Value
Non-significant difference in gestational age (p>0.05)	Botet 2011	0.78	0.51	1.21	0.272
	Barrera-Reyes 2011	1.09	0.42	2.83	0.855
	Bry 2015	1.71	0.09	31.92	0.718
	Ohyama 2002	2.24	0.98	5.10	0.056
	Honma 2007	2.60	1.14	5.93	0.023
	Misra 2015	2.85	0.78	10.47	0.114
	**Non-significant difference**	**1.54**	**0.88**	**2.70**	**0.127**
Significant difference in gestational age(p<0.05)	Prendergast 2011	0.63	0.29	1.36	0.238
	Soraisham 2013	0.70	0.44	1.12	0.135
	Thomas 2009	0.73	0.21	2.56	0.627
	Miralles 2008	1.16	0.24	5.53	0.856
	van Vliet 2012	1.23	0.43	3.54	0.700
	Dempsey 2005	1.35	0.60	3.02	0.464
	Richardson 2006	1.50	1.02	2.20	0.038
	Sato 2011	1.57	0.99	2.50	0.056
	Gray 1997	1.81	0.63	5.26	0.273
	Baud 2000	1.92	1.33	2.77	0.001
	Rocha 2006	2.45	1.16	5.19	0.019
	Miyazaki 2016	2.63	2.29	3.01	0.000
	Mu 2008	2.88	1.23	6.75	0.015
	Wirbelauer 2011	3.21	1.04	9.95	0.043
	Lee Yeri 2015	3.93	1.92	8.03	0.000
	Lee Hyun Ju 2011	4.87	1.39	17.07	0.013
	Watterberg 1996	6.11	1.20	31.16	0.029
	Xie 2015	6.65	0.87	50.95	0.068
	Jones 2013	10.96	0.62	193.72	0.102
	De Felice 2005	15.16	4.77	48.18	0.000
	**Significant difference**	**2.00**	**1.49**	**2.69**	**0.000**
	**OVERALL**	**1.89**	**1.46**	**2.45**	**0.000**

Heterogeneity: $p < 0.001$; $I^2 = 73\%$

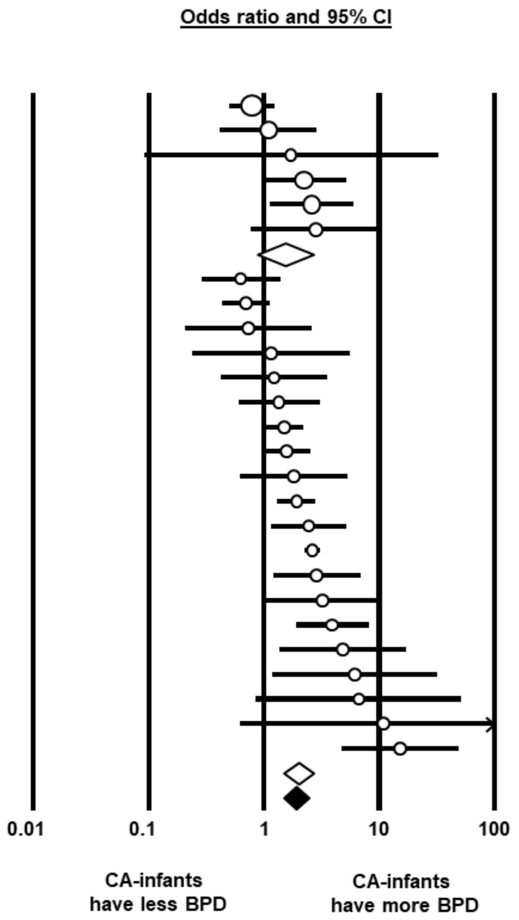

BPD: bronchopulmonary dysplasia; BPD28: bronchopulmonary dysplasia defined as supplemental oxygen requirement on postnatal day 28; CA: chorioamnionitis; CI: confidence interval.

© 2019 Villamor-Martinez E et al. *JAMA Network Open.*

eFigure 15. Meta-Analysis of Chorioamnionitis and BPD36, Grouped by Significant/Nonsignificant Difference in Gestational Age

Subgroup	Study name	Odds ratio	Lower limit	Upper limit	p-Value
Non-significant difference in GA (p>0.05)	Nasef (c) 2013	0.39	0.13	1.19	0.098
	Young 2005	0.43	0.23	0.81	0.010
	Schlapbach 2010	0.50	0.13	1.93	0.315
	Botet 2011	0.62	0.33	1.17	0.139
	Nasef (h) 2013	0.81	0.44	1.48	0.489
	Nicaise 2002	0.93	0.23	3.83	0.923
	Trevisanuto 2013	0.94	0.36	2.47	0.896
	Ahn 2012	0.98	0.55	1.76	0.952
	Arayici 2014	1.10	0.49	2.48	0.810
	Fung 2003	1.56	0.46	5.31	0.481
	Gonzáles-Luis 2002	1.63	0.63	4.20	0.317
	Stepan 2016	1.69	0.08	36.08	0.739
	Seliga-Sizecka 2013	1.75	1.00	3.08	0.051
	Misra 2015	2.25	0.36	13.97	0.384
	Bry 2015	2.67	0.43	16.39	0.290
	**Non-significant difference**	**0.92**	**0.70**	**1.23**	**0.588**
Significant difference in GA (p<0.05)	Miralles 2008	0.29	0.03	2.98	0.299
	Thomas 2009	0.57	0.10	3.22	0.520
	Plakkal 2013	0.58	0.38	0.89	0.012
	Kewitz 2008	0.70	0.32	1.54	0.373
	Bordigato 2011	0.80	0.16	3.88	0.782
	Prendergast 2011	0.85	0.39	1.81	0.668
	Watterberg 1999	0.90	0.25	3.25	0.870
	Pappas (h) 2014	0.92	0.77	1.11	0.391
	Pappas (Both) 2014	1.04	0.83	1.29	0.759
	Kent 2004	1.04	0.59	1.83	0.897
	Suppiej 2009	1.08	0.46	2.52	0.867
	Strunk 2012	1.11	0.82	1.48	0.505
	Been 2010	1.23	0.65	2.33	0.529
	Soraisham 2009	1.39	1.11	1.74	0.004
	Gagliardi 2014	1.41	1.21	1.64	0.000
	Kim 2015	1.50	0.83	2.72	0.178
	Hendson 2011	1.55	1.07	2.23	0.019
	Liu 2014	1.60	0.58	4.36	0.362
	Lee Hyun Ju 2011	1.61	0.87	2.98	0.129
	Garcia-Munoz Rodrigo 2014	1.72	1.50	1.98	0.000
	Lau 2005	1.88	1.32	2.66	0.000
	Ogunyemi 2003	2.03	1.40	2.94	0.000
	Zanardo 2008	2.19	1.16	4.13	0.015
	Hitti 2001	2.28	0.72	7.22	0.161
	Polam 2005	2.38	1.26	4.49	0.008
	Mestan 2010	3.04	1.26	7.35	0.013
	Curley 2003	3.24	1.09	9.58	0.034
	Choi 2008	3.81	1.27	11.39	0.017
	Perrone 2012	3.82	1.54	9.44	0.004
	Natarajan 2008	3.96	0.93	16.90	0.063
	Erdemir 2013	21.50	3.50	131.90	0.001
	**Significant difference**	**1.43**	**1.23**	**1.67**	**0.000**
	**OVERALL**	**1.30**	**1.13**	**1.48**	**0.000**

Heterogeneity: $p < 0.001$; $I^2 = 66\%$

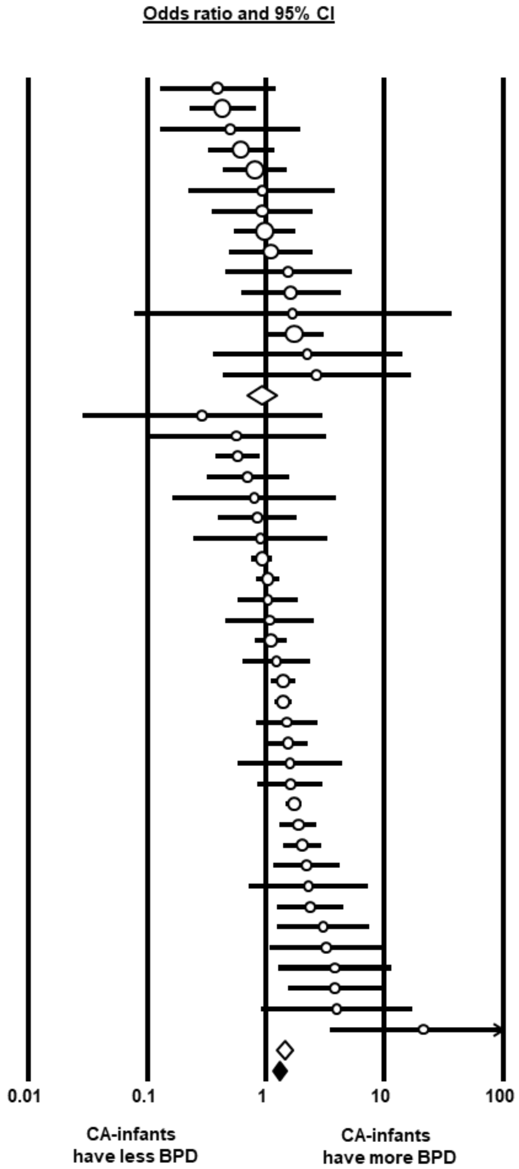

BPD: bronchopulmonary dysplasia; BPD36: bronchopulmonary dysplasia, defined as supplemental oxygen requirement at the postmenstrual age of 36 weeks; CA: chorioamnionitis; CI: confidence interval.

© 2019 Villamor-Martinez E et al. *JAMA Network Open.*

eFigure 16. Funnel Plots Assessing Publication Bias for the Association Between Chorioamnionitis and Bronchopulmonary Dysplasia

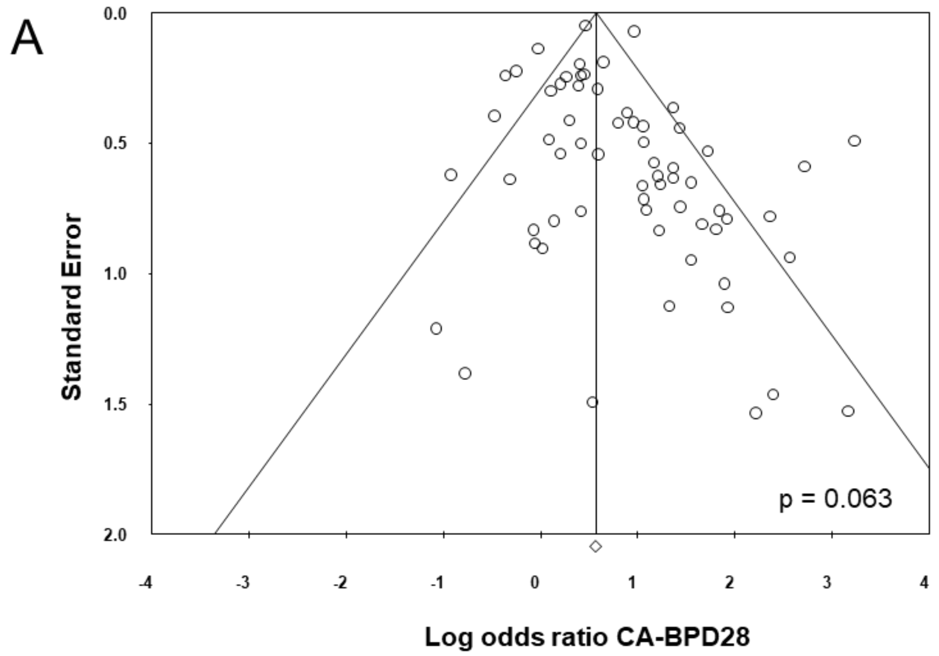

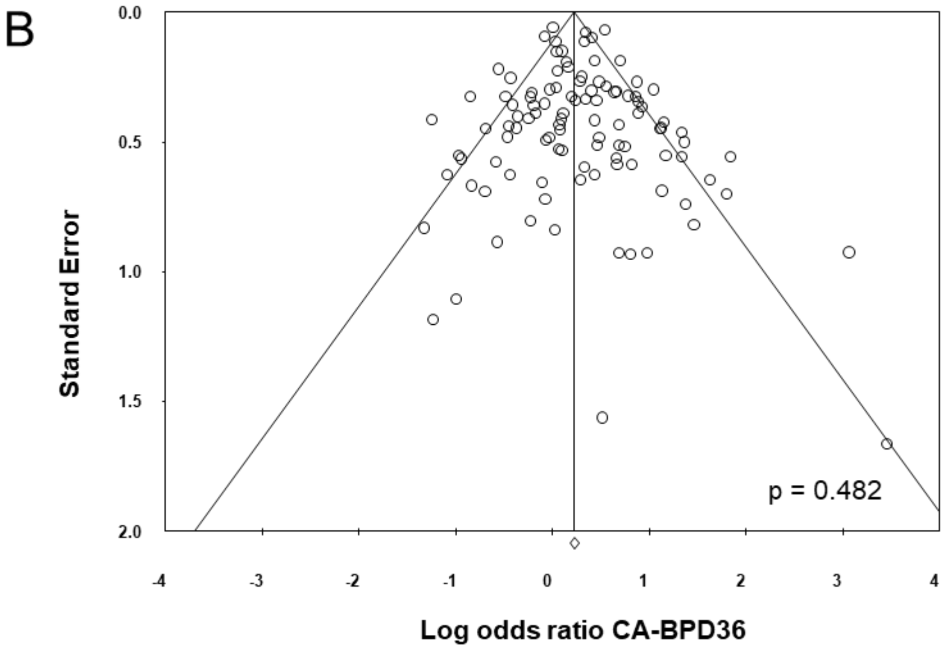

A. Chorioamnionitis and BPD28. B. Chorioamnionitis and BPD36.

BPD36: bronchopulmonary dysplasia defined as supplemental oxygen requirement at the postmenstrual age of 36 weeks; BPD28: bronchopulmonary dysplasia defined as supplemental oxygen requirement on postnatal day 28.

© 2019 Villamor-Martinez E et al. *JAMA Network Open.*

eTable 1. Characteristics of All Included Studies

First author, Year	Country	Prospective /Retrospective	Cohort /Case-control	Perspective	Total infants (centers)	Max GA	Max BW	Definition CA	Definition BPD	NOS Score
Abele-Horn 1997	Germany	Prospective	Cohort	CA	170 (1)	PROM[1]	N/A	Microbiological	BPD28	8
Abele-Horn 1998	Germany	Prospective	Cohort	CA	97 (2)	N/A	1499	Microbiological	BPD28 & BPD36	9
Ahn 2012	South Korea	Prospective	Cohort	CA	257 (1)	33 6/7	N/A	Histological	BPD36	9
Alshehri 2014	Saudi Arabia	Retrospective	Cohort	BPD	942 (1)	31 6/7	1499	Unspecified	BPD28 & BPD36	7
Ameenudeen 2007	Malaysia	Prospective	Cohort	BPD	236 (1)	N/A	1499	Clinical	BPD36	9
Arayici 2014	Turkey	Retrospective	Cohort	CA	281 (1)	32	1500	Histological	BPD36	8
Bagchi 1994	USA	Prospective	Case-control	BPD	40 (1)	32 6/7	N/A	Unspecified	BPD28	6
Baier 2003	USA	Prospective	Cohort	BPD	24 (1)	N/A	1499	Unspecified	BPD28 & BPD36	7
Baker 2012	USA	Prospective	Cohort	BPD	62 (1)	35 6/7	N/A	Clinical	BPD36	8
Barrera-Reyes 2011	Mexico	Prospective	Cohort	CA	104 (1)	34	1500	Clinical	BPD28 & BPD36	8
Baud 2000	France	Prospective	Cohort	CA	685 (1)	32 6/7	N/A	Clinical	BPD36	9
Been 2010	Netherlands	Prospective	Cohort	CA	301 (1)	32	N/A	Histological	BPD28 & BPD36	8
Bordigato 2011	Italy	Prospective	Cohort	CA	29 (1)	27 6/7	999	Histological	BPD28 & BPD36	8
Bose 2011	USA	Prospective	Cohort	BPD	932 (14)	27 6/7	N/A	Histological	BPD28 & BPD36	8
Botet 2011	Spain	Prospective	Case-control	CA	328 (12)	N/A	1499	Clinical	BPD36	8
Brener Dick 2017	Argentina	Retrospective	Cohort	BPD	115 (1)	N/A	1499	Unspecified	BPD36	7
Bry 2015	Sweden	Prospective	Cohort	CA	21 (1)	28 6/7	N/A	Histological	BPD28	8
Cederqvist 2003	Finland	Prospective	Cohort	BPD	32 (1)	36 6/7	N/A	Clinical	BPD28	7
Chisholm 2016	USA	Prospective	Cohort	CA	102 (1)	34	2000	Histological	BPD36	8

[1] All mothers of included infants had PROM

© 2019 Villamor-Martinez E et al. *JAMA Network Open.*

Choi 2005	South Korea	Prospective	Cohort	BPD	115 (1)	N/A	1499	Histological	BPD28	7
Choi 2006	South Korea	Prospective	Cohort	BPD	75 (1)	32	N/A	Histological	BPD28	8
Choi 2008	South Korea	Prospective	Cohort	CA	63 (2)	33 6/7	N/A	Histological	BPD36	9
Colaizy 2007	USA	Prospective	Cohort	BPD	121 (1)	N/A	1499	Clinical	BPD36	8
Curley 2003	UK	Prospective	Cohort	CA	79 (1)	32 6/7	N/A	Histological	BPD36	8
De Felice 2004	Italy	Retrospective	Case-control	BPD	30 (1)	GA-matched[2]	N/A	Histological	BPD28	8
De Felice 2005	Italy	Prospective	Cohort	CA	103 (1)	N/A	1499	Histological	BPD28	8
Demirel 2009	Turkey	Retrospective	Case-control	BPD	106 (1)	N/A	1500	Unspecified	BPD36	7
Dempsey 2005	Canada	Retrospective	Cohort	CA	330 (1)	29 6/7	N/A	Histological	N/A	7
Dessardo 2014	Croatia)	Prospective	Cohort	CA	262 (1)	31 6/7	N/A	Histological	BPD36	7
Dexter 1999	USA	Retrospective	Cohort	CA	317 (1)	N/A	1249	Clinical	BPD28	8
Dexter 2000	Turkey	Prospective	Cohort	CA	275 (1)	N/A	1249	Histological	BPD28	8
Durrmeyer 2012	France	Prospective	Cohort	BPD	258 (1)	26 6/7	N/A	Histological	BPD36	8
Ecevit 2014	Turkey	Retrospective	Cohort	CA	19 (1)	36 6/7	N/A	Histological	BPD28	8
Erdemir 2013	Turkey	Prospective	Cohort	CA	57 (1)	34 6/7	N/A	Histological	BPD36	8
Eriksson 2014	Sweden	Retrospective	Cohort	BPD	106339 (N/A)	36 6/7	N/A	Unspecified	BPD28	8
EXPRESS group 2010	Sweden	Prospective	Cohort	BPD	638 (N/A)	26 6/7	N/A	Clinical	BPD36	8
Fujioka 2014	Japan	Retrospective	Cohort	BPD	92 (1)	31 6/7	N/A	Histological	BPD28	8
Fukunaga 2009	Medicine	Retrospective	Cohort	BPD	29 (1)	29 6/7	N/A	Histological	BPD36	8
Fung 2003	Hong Kong	Both	Cohort	CA	62 (1)	27 6/7	999	Microbiological	BPD28 & BPD36	8
Gagliardi 2014	Italy	Prospective	Cohort	CA	3606 (82)	29 6/7	1500	Clinical	BPD36	8
Gantar 2011	Slovenia	Prospective	Cohort	BPD	65	29 6/7	N/A	Both defined	BPD36	8
Garcia-Munoz Rodrigo 2014	Spain	Prospective	Cohort	CA	8330 (53)	32	1499	Clinical	BPD36	9
Ghezzi 1998	USA	Prospective	Cohort	BPD	47 (1)	27 6/7	N/A	Histological	BPD28 & BPD36	8
Gonzáles-Luis 2002	Spain	Retrospective	Case-control	CA	135 (1)	N/A	1499	Clinical	BPD36	8

[2] Preterm infants with BPD were enrolled. Control infants without BPD were matched by GA and sex.

© 2019 Villamor-Martinez E et al. *JAMA Network Open.*

Gray 1997	Australia	Both	Cohort	CA	158 (1)	29 6/7	N/A	Clinical	BPD28	8
Guimaraes 2010	Portugal	Prospective	Cohort	BPD	256 (5)	29 6/7	1249	Clinical	BPD36	8
Guo 2015	Turkey	Retrospective	Cohort	BPD	75 (1)	31 6/7	1499	Unspecified	BPD28 & BPD36	7
Hansen 2010	USA	Prospective	Cohort	BPD	107 (1)	31 6/7	N/A	Clinical	BPD36	8
Hendson 2011	Canada	Prospective	Cohort	CA	507 (1)	32	1250	Histological	BPD28	8
Hikino 2012	Japan	Prospective	Cohort	BPD	26 (1)	33 6/7	N/A	Histological	BPD28	8
Hitti 2001	Sweden	Prospective	Cohort	CA	140 (3)	34	N/A	Microbiological	BPD36	8
Honma 2007	Japan	Retrospective	Cohort	CA	105 (1)	31 6/7	N/A	Histological	BPD28	8
Hyödynmaa 2012	Finland	Prospective	Cohort	BPD	82 (1)	N/A	1500	Unspecified	BPD28	7
Ikeda 2015	Japan	Prospective	Cohort	BPD	290 (1)	31 6/7	N/A	Histological	BPD28 & BPD36	8
Iwatani 2013	Japan	Retrospective	Cohort	BPD	51 (1)	27 6/7	N/A	Histological	BPD28	8
Jones 2013	Brazil	Prospective	Cohort	CA	95 (1)	36 6/7	N/A	Histological	BPD28	8
Jonsson 1998	Sweden.	Prospective	Cohort	CA	(1)	29 6/7	N/A	Clinical	BPD28 & BPD36	9
Kalra 2014	USA	Prospective	Case-control	BPD	60 (2)	32	N/A	Histological	BPD36	8
Kandasamy (h) 2015	USA	Prospective	Cohort	BPD	152 (1)	N/A	999	Both defined	BPD36	8
Karagianni 2011	Greece	Prospective	Cohort	BPD	219 (1)	32	1500	Unspecified	BPD28 & BPD36	7
Karagianni 2013	Greece	Prospective	Case-control	BPD	61 (1)	31 6/7	N/A	Unspecified	BPD28	7
Kaukola 2009	Finland	Prospective	Cohort	CA	82 (1)	31 6/7	N/A	Histological	BPD36	8
Kazzi 2004	USA	Prospective	Cohort	BPD	120 (1)	N/A	1250	Unspecified	BPD36	7
Kent 2004	Australia	Prospective	Cohort	CA	241 (1)	29 6/7	N/A	Histological	BPD28 & BPD36	9
Kewitz 2008	Germany	Retrospective	Cohort	CA-BPD	115 (1)	27 6/7	N/A	Histological	BPD36	7
Khan 2006	USA	Retrospective	Cohort	BPD	306 (2)	29 6/7	N/A	Microbiological	BPD36	8
Kim 2004	South Korea	Retrospective	Cohort	CA-BPD	478 (1)	34	N/A	Histological	BPD36	8
Kim 2008	South Korea	Prospective	Case-control	BPD	74 (1)	31 6/7	N/A	Histological	BPD36	8
Kim 2015	South Korea	Retrospective	Cohort	CA	234 (1)	N/A	1499	Histological	BPD36	9
Kirchner 2007	Austria	Retrospective	Cohort	CA	44 (1)	N/A	1449	Microbiological	BPD36	8
Klinger 2013	Israel	Prospective	Cohort	BPD	12139 (30)	N/A	1500	Clinical	BPD36	8

© 2019 Villamor-Martinez E et al. *JAMA Network Open.*

Koroglu 2013	Turkey	Prospective	Case-control	BPD	41 (1)	31 6/7	N/A	Clinical	BPD28	8
Lahra 2009	Australia	Prospective	Cohort	BPD	761 (1)	29 6/7	N/A	Histological	BPD36	8
Lamboley-Gilmert 2003	France	Prospective	Cohort	BPD	648 (33)	32 6/7	N/A	Microbiological	BPD28 & BPD36	8
Lapcharoensap 2015	USA	Retrospective	Cohort	BPD	15052 ()	29 6/7	1500	Clinical	BPD36	8
Lardon-Fernandez 2017	Spain	Retrospective	Cohort	BPD	129 (1)	N/A	1499	Clinical	BPD36	8
Lau 2005	Turkey	Prospective	Cohort	CA	1296 (1)	N/A[3]	N/A	Histological	BPD36	8
Lee Hyun Ju 2011	South Korea	Retrospective	Cohort	CA	147 (2)	N/A	999	Histological	BPD28 & BPD36	8
Lee Yeri 2015	South Korea	Retrospective	Cohort	CA	339 (1)	33 6/7	N/A	Histological	BPD28	8
Leroy 2018	Canada	Prospective	Cohort	BPD	62 (1)	30	N/A	Histological	BPD36	8
Li 2015	China	Retrospective	Cohort	BPD	47 (N/A)	36 6/7	N/A	Unspecified	BPD28 & BPD36	6
Lin 2005	China	Prospective	Case-control	BPD	224 (1)	29 6/7	N/A	Clinical	BPD36	8
Liu 2014	China	Prospective	Cohort	CA	216 (1)	33 6/7	N/A	Histological	BPD36	8
Lodha 2014	Canada	Prospective	Cohort	BPD	1030 (1)	N/A	1250	Unspecified	BPD36	7
Lohmann 2014	USA	Prospective	Cohort	BPD	22 (1)	32	N/A	Histological	BPD36	8
Mahlman 2017	Finland	Prospective	Cohort	BPD	174 (5)	30 6/7	N/A	Clinical	BPD28 & BPD36	8
Mailaparambil 2010	Germany	Prospective	Cohort	BPD	155 (1)	28	N/A	Histological	BPD28	8
May 2011	UK	Prospective	Cohort	BPD	44 (1)	32 6/7	N/A	Histological	BPD28	8
McGowan (h) 2009	Turkey	Retrospective	Case-control	BPD	96 (1)	N/A	1250	Both defined	N/A	8
Mehta 2006	USA	Retrospective	Cohort	CA	164 (1)	33 6/7	N/A	Histological	BPD36	8
Mestan 2010	USA	Prospective	Case-control	CA	202 (1)	36 6/7	N/A	Histological	BPD36	8
Metcalfe 2017	USA	Retrospective	Cohort	CA-BPD	53906	36	N/A	Clinical	BPD28	9
Miralles 2008	UK	Prospective	Cohort	CA	31 (1)	31 6/7	N/A	Microbiological	BPD28 & BPD36	8
Misra 2015	USA	Retrospective	Cohort	CA	40 (1)	32	N/A	Histological	BPD36	8
Mittendorf 2005	USA	Prospective	Cohort	BPD	123 (1)	33 6/7	N/A	Histological	BPD28	8
Miyazaki 2016	Japan	Retrospective	Cohort	CA	4078 (N/A)	33 6/7	N/A	Histological	BPD36	9

[3] Infants admitted to NICU were included.

© 2019 Villamor-Martinez E et al. *JAMA Network Open.*

Morrow 2017	USA	Prospective	Cohort	BPD	587 (5)	34	1250	Unspecified	BPD36	7
Mu 2008	Taiwan	Prospective	Cohort	CA	119 (1)	N/A	1499	Histological	N/A	8
Nasef (h) 2013	Turkey	Retrospective	Cohort	CA	241 (1)	29 5/7	N/A	Both defined	BPD36	8
Natarajan 2008	USA	Prospective	Cohort	CA	48 (1)	N/A	1499	Histological	BPD36	8
Nicaise 2002	France	Prospective	Cohort	CA	112 (1)	N/A[4]	N/A	Histological	BPD36	7
Nishimaki 2003	Japan	Prospective	Cohort	CA	69 (1)	32	N/A	Histological	BPD28	8
Novitsky 2015	Usa	Retrospective	Cohort	BPD	906 (1)	N/A	1499	Clinical	BPD36	8
Ogunyemi 2003	Usa	Retrospective	Cohort	CA	774 (1)	31 6/7	N/A	Histological	BPD36	8
Oh 2015	South Korea	Prospective	Cohort	CA	175 (1)	N/A	999	Histological	BPD36	8
Ohyama 2002	Japan	Retrospective	Cohort	CA	143 (1)	31 6/7	N/A	Histological	BPD36	9
O'Shea 1998	USA	Prospective	Cohort	CA	70 (1)	N/A	1499	Histological	BPD28 & BPD36	8
Paananen 2009	Finland	Prospective	Cohort	CA-BPD	123 (1)	N/A	1499	Histological	BPD36	9
Pappas (h) 2014	USA	Prospective	Cohort	CA	1924 (16)	26 6/7	N/A	Both defined	BPD36	8
Perrone 2012	Italy	Prospective	Cohort	CA	92 (1)	31 6/7	N/A	Histological	BPD36	8
Plakkal 2013	Canada	Retrospective	Cohort	CA	444 (1)	28 6/7	N/A	Histological	BPD36	9
Polam 2005	USA	Prospective	Cohort	CA	177 (1)	28 6/7	N/A	Histological	BPD36	8
Prendergast 2011	UK	Retrospective	Cohort	CA	120 (1)	32	N/A	Histological	BPD36	8
Redline (h) 2002	USA	Prospective	Cohort	CA	371 (1)	31 6/7	1499	Both defined	BPD36	9
Richardson 2006	Turkey	Prospective	Cohort	CA	660 (1)	33 6/7	N/A	Histological	BPD28	8
Rindfleisch 1996	USA	Prospective	Cohort	BPD	36 (1)	32 6/7	N/A	Clinical	BPD28	8
Rocha 2006	Portugal	Retrospective	Cohort	CA	452 (3)	33 6/7	N/A	Histological	BPD28	9
Rocha 2010	Portugal.	Retrospective	Cohort	BPD	205 (1)	31 6/7	1249	Histological	BPD36	9
Rocha 2011	Portugal	Prospective	Cohort	BPD	156 (2)	31 6/7	N/A	Histological	BPD36	8
Rojas 2012	Colombia	Retrospective	Case-control	BPD	212 (8)	30 6/7	N/A	Microbiological	BPD28 & BPD36	8
Sampath 2015	USA	Prospective	Cohort	BPD	659 (5)	N/A	1500	Clinical	BPD36	8
Sato 2011	Japan	Retrospective	Cohort	CA	302 (1)	29 6/7	N/A	Histological	BPD28	8

[4] All mothers of included infants had PROM

© 2019 Villamor-Martinez E et al. *JAMA Network Open.*

Schena 2015	Italy	Retrospective	Cohort	BPD	242 (1)	28	N/A	Clinical	BPD28	8
Schlapbach 2010	Switzerland	Prospective	Case-control	CA	99 (1)	31 6/7	N/A	Both	BPD36	8
Seliga-Sizecka 2013	Poland	Prospective	Cohort	CA	383 (1)	31 6/7	N/A	Histological	BPD36	8
Serenius 2004	Sweden	Retrospective	Cohort	BPD	(2)	25 6/7	N/A	Unspecified	BPD36	7
Shima 2011	Japan	Retrospective	Cohort	BPD	306 (1)	31 6/7	N/A	Histological	BPD28 & BPD36	8
Shima 2013	Japan	Prospective	Cohort	BPD	96 (1)	27 6/7	N/A	Histological	BPD36	8
Smit 2015	Netherlands	Prospective	Cohort	CA	300 (1)	32	N/A	Histological	BPD36	8
Soliman 2017	Canada	Prospective	Cohort	BPD	319 (1)	31 6/7	N/A	Histological	BPD36	8
Soraisham 2009	Canada	Prospective	Cohort	CA	3094 (24)	32 6/7	N/A	Histological	BPD36	9
Soraisham 2013	Canada	Retrospective	Cohort	CA	384 (1)	28 6/7	N/A	Histological	BPD28 & BPD36	8
Stepan 2016	Czech Republic	Prospective	Cohort	CA	122 (1)	34	N/A	Histological	BPD36	8
Stichel 2011	Sweden	Prospective	Cohort	BPD	51 (1)	28 6/7	N/A	Clinical	BPD36	8
Stimac 2014	Croatia	Retrospective	Cohort	CA	395 (1)	N/A	1499	Histological	BPD28	8
Streubel 2008	USA	Retrospective	Cohort	BPD	133 (1)	30 6/7	999	Histological	BPD36	8
Strunk 2012	Australia	Retrospective	Cohort	CA	838 (1)	29 6/7	N/A	Histological	BPD28	8
Suppiej 2009	Italy	Prospective	Cohort	CA	104 (1)	31 6/7	N/A	Histological		7
Thomas 2009	Poland	Prospective	Cohort	CA	42 (1)	29 6/7	1499	Histological	BPD28 & BPD36	8
Tokuriki 2015	Japan	Prospective	Cohort	BPD	25 (1)	32 6/7	1499	Unspecified	BPD28	7
Torchin 2017	France	Prospective	Cohort	CA-BPD	1731	31	N/A	Histological	BPD36	9
Trevisanuto 2013	Italy	Prospective	Case-control	CA	98 (1)	31 6/7	N/A	Histological	BPD36	8
Tsiartas 2013	Czech Republic	Both	Cohort	CA	231 (1)	36 6/7	N/A	Histological	BPD28 & BPD36	8
Van Marter 2002	USA	Prospective	Case-control	CA	257 (3)	N/A	1500	Histological	BPD36	9
van Vliet 2012	Netherlands	Prospective	RCT	CA	72 (1)	31 6/7	1499	Histological	BPD28	9
Viscardi 2004	Turkey	Prospective	Cohort	BPD	(2)	32 6/7	1500	Histological	BPD28 & BPD36	9
Wang 2014	China	Prospective	Cohort	BPD	73 (1)	32	1500	Clinical	BPD28	8
Watterberg 1996	Turkey	Prospective	Cohort	CA	30 (1)	N/A	1999	Histological	BPD28 & BPD36	8

© 2019 Villamor-Martinez E et al. *JAMA Network Open.*

Watterberg 1999	USA	Prospective	RCT	CA	40 (2)	N/A	999	Histological	BPD28	8
Watterberg 2004	USA	Prospective	RCT	BPD	360 (>3)	N/A	999	Histological	BPD36	9
Wirbelauer 2011	Germany	Prospective	Cohort	CA	71 (1)	N/A	1499	Histological	BPD28	8
Xie 2015	China	Retrospective	Cohort	CA	371 (1)	33 6/7	N/A	Histological	N/A	7
Xie 2016	China	Prospective	Cohort	BPD	35 (1)	29 6/7	1500	Histological	BPD28	8
Yoon 1999	South Korea	Prospective	Cohort	BPD	171 (1)	33 6/7	N/A	Histological	BPD28	8
Young 2005	Turkey	Retrospective	Cohort	CA	308 (1)	31 6/7	999	Histological	BPD28 & BPD36	8
Zanardo 2002	Italy	Retrospective	Case-control	BPD	100 (1)	31	N/A	Histological	BPD28	8
Zanardo 2008	Italy	Prospective	Cohort	CA	287 (1)	31 6/7	N/A	Histological	BPD36	8
Zhang 2011	China	Retrospective	Cohort	BPD	116 (1)	N/A	1500	Histological	BPD28	8

GA: gestational age; BW: birth weight; CA: chorioamnionitis; BPD: bronchopulmonary dysplasia; BPD28: bronchopulmonary dysplasia defined as oxygen dependency at 28 days of life; BPD36: bronchopulmonary dysplasia defined as oxygen dependency at 36 weeks post-menstrual age; RCT: randomized controlled trial.

Perspective CA: studies looked at the outcomes of preterm infants with and without CA and BPD was one of these outcomes.

Perspective BPD: studies analyzed risk factors for BPD, and CA was one of these risk factors.

Perspective CA-BPD: studies were designed to primarily examine the association between CA and BPD.

© 2019 Villamor-Martinez E et al. *JAMA Network Open.*

eTable 2. Meta-Regression Analyses of Risk of BPD and Covariates

BPD def.	Meta-regression	k	CC	95% CI	Z	P	R^2
BPD28	Gestational age (MD, weeks)	26	-0.50	-0.75 to 0.25	-3.88	<**0.001**	0.71
	Birth weight (MD, per 100 g)	26	-0.07	-0.21 to 0.07	-1.01	0.313	0.00
	CA type (histological/clinical)	62	-0.38	-0.84 to 0.09	-1.58	0.114	0.00
	ACS (log OR)	20	0.21	-0.11 to 0.53	1.28	0.201	0.35
	Cesarean section (log OR)	16	-0.07	-0.44 to 0.30	-0.37	0.708	0.00
	Maternal age (MD, years)	11	-0.06	-0.49 to 0.38	-0.26	0.796	0.12
	SGA (log OR)	10	-0.13	-1.01 to 0.75	-0.29	0.770	0.00
	PROM (log OR)	14	-0.46	-1.12 to 0.20	-1.37	0.172	0.00
	Mortality (log OR)	18	0.09	-0.13 to 0.31	0.81	0.416	0.00
	Early onset sepsis (log OR)	12	0.05	-0.31 to 0.42	0.28	0.781	0.00
	Late onset sepsis (log OR)	12	0.05	-0.15 to 0.25	0.45	0.654	0.00
	RDS (log OR)	24	0.10	-0.02 to 0.23	1.64	0.100	0.11
BPD36	Gestational age (MD, weeks)	48	-0.22	-0.33 to -0.12	-4.07	<**0.001**	0.39
	Birth weight (MD, per 100 g)	44	-0.32	-0.45 to -0.20	-5.03	<**0.001**	0.41
	CA type (histological/clinical)	99	-0.07	-0.29 to 0.15	-0.64	0.521	0.00
	ACS (log OR)	38	0.02	-0.27 to 0.30	0.10	0.918	0.00
	Cesarean section (log OR)	26	0.00	-0.18 to 0.18	0.04	0.969	0.00
	Maternal age (MD)	17	-0.26	-0.56 to 0.05	-1.65	0.098	0.00
	SGA (log OR)	14	0.12	-0.22 to 0.45	0.69	0.493	0.00
	PROM (log OR)	28	-0.07	-0.15 to 0.01	-1.76	0.079	0.00
	Preeclampsia (log OR)	16	0.09	-0.11 to 0.29	0.88	0.379	0.00
	Mortality (log OR)	27	0.07	-0.20 to 0.33	0.50	0.614	0.00
	Early onset sepsis (log OR)	24	-0.02	-0.13 to 0.09	-0.35	0.724	0.03
	Late onset sepsis (log OR)	26	0.25	-0.09 to 0.58	1.46	0.145	0.00
	RDS (log OR)	29	0.36	0.19 to 0.53	4.16	<**0.001**	0.47

BPD28: bronchopulmonary dysplasia defined as oxygen dependency at 28 days of life; BPD36: bronchopulmonary dysplasia defined as oxygen dependency at 36 weeks post-menstrual age; k: number of studies included in analysis; CC: coefficient; CI: confidence interval; MD: mean difference; CA: chorioamnionitis; OR: odds ratio; ACS: antenatal corticosteroids; SGA: small for gestational age; RDS: respiratory distress syndrome. **Values marked in bold: p <0.05.** Note: Analysis of CA associated risk of BPD28 and preeclampsia was not carried out due to a lack of studies.

© 2019 Villamor-Martinez E et al. *JAMA Network Open.*

eTable 3. Newcastle-Ottawa Quality Assessment of Included Studies

First author, year	Selection	Compara	Outcome	Total	Reasons for downgrade
Abele-Horn 1997	4	1	3	8	No adjustment for confounders
Abele-Horn 1998	4	2	3	9	
Ahn 2012	4	2	3	9	
Alshehri 2014	3	1	3	7	No CA definition, No adjustment for confounders
Ameenudeen 2007	4	2	3	9	
Arayici 2014	4	1	3	8	No adjustment for confounders
Bagchi 1994	2	1	3	6	No CA definition, Non-representative population (all infants intubated), No adjustment for confounders
Baier 2003	3	1	3	7	No CA definition, No adjustment for confounders
Baker 2012	4	1	3	8	No adjustment for confounders
Barrera-Reyes 2011	4	1	3	8	No adjustment for confounders
Baud 2000	4	2	3	9	
Been 2010	4	1	3	8	No adjustment for confounders
Bordigato 2011	4	1	3	8	No adjustment for confounders
Bose 2011	4	1	3	8	No adjustment for confounders
Botet 2011	4	1	3	8	No adjustment for confounders
Brener Dick 2017	3	1	3	7	No CA definition, No adjustment for confounders
Bry 2015	4	1	3	8	No adjustment for confounders
Cederqvist 2003	3	1	3	7	No adjustment for confounders
Chisholm 2016	4	1	3	8	No adjustment for confounders
Choi 2005	4	1	2	7	No adjustment for confounders
Choi 2006	4	1	3	8	No adjustment for confounders
Choi 2008	4	2	3	9	
Colaizy 2007	4	1	3	8	No adjustment for confounders
Curley 2003	4	1	3	8	No adjustment for confounders
De Felice 2004	4	1	3	8	No adjustment for confounders
De Felice 2005	4	1	3	8	No adjustment for confounders
Demirel 2009	3	1	3	7	No CA definition, No adjustment for confounders
Dempsey 2005	4	1	2	7	No adjustment for confounders, BPD not defined
Dessardo 2014	4	1	2	7	No adjustment for confounders
Dexter 1999	4	1	3	8	No adjustment for confounders
Dexter 2000	4	1	3	8	No adjustment for confounders
Durrmeyer 2012	4	1	3	8	No adjustment for confounders
Ecevit 2014	4	1	3	8	No adjustment for confounders
Erdemir 2013	4	1	3	8	No adjustment for confounders
Eriksson 2014	3	2	3	8	No CA definition
EXPRESS group 2010	4	1	3	8	No adjustment for confounders

© 2019 Villamor-Martinez E et al. *JAMA Network Open.*

Fujioka 2014	4	1	3	8	No adjustment for confounders
Fukunaga 2009	4	1	3	8	No adjustment for confounders
Fung 2003	4	1	3	8	No adjustment for confounders
Gagliardi 2014	4	1	3	8	No adjustment for confounders
Gantar 2011 (h)	4	1	3	8	No adjustment for confounders
Garcia-Munoz Rodrigo 2014	4	2	3	9	
Ghezzi 1998	4	1	3	8	No adjustment for confounders
Gonzáles-Luis 2002	4	1	3	8	No adjustment for confounders
Gray 1997	4	1	3	8	No adjustment for confounders
Guimaraes 2010	4	1	3	8	No adjustment for confounders
Guo 2015	3	1	3	7	No CA definition, No adjustment for confounders
Hansen 2010	4	1	3	8	No adjustment for confounders
Hendson 2011	4	1	3	8	No adjustment for confounders
Hikino 2012	4	1	3	8	No adjustment for confounders
Hitti 2001	4	1	3	8	No adjustment for confounders
Honma 2007	4	1	3	8	No adjustment for confounders
Hyodynmaa 2012	3	1	3	7	No CA definition, No adjustment for confounders
Ikeda 2015	4	1	3	8	No adjustment for confounders
Iwatani 2013	4	1	3	8	No adjustment for confounders
Jones 2013	4	1	3	8	No adjustment for confounders
Jonsson 1998	4	2	3	9	
Kalra 2014	4	1	3	8	No adjustment for confounders
Kandasamy (h) 2015	4	1	3	8	No adjustment for confounders
Karagianni 2011	3	1	3	7	No CA definition, No adjustment for confounders
Karagianni 2013	3	1	3	7	No CA definition, No adjustment for confounders
Kaukola 2009	4	1	3	8	No adjustment for confounders
Kazzi 2004	3	1	3	7	No CA definition, No adjustment for confounders
Kent 2004	4	2	3	9	
Kewitz 2008	3	1	3	7	Non-representative population (SGA infants excluded), No adjustment for confounders
Khan 2006	4	1	3	8	No adjustment for confounders
Kim 2004	4	1	3	8	No adjustment for confounders
Kim 2008	4	1	3	8	No adjustment for confounders
Kim 2015	4	2	3	9	
Kirchner 2007	4	1	3	8	No adjustment for confounders
Klinger 2013	4	1	3	8	No adjustment for confounders
Koroglu 2013	4	1	3	8	No adjustment for confounders
Lahra 2009	4	1	3	8	No adjustment for confounders
Lamboley-Gilmert 2003	4	1	3	8	No adjustment for confounders
Lapcharoensap 2015	4	1	3	8	No adjustment for confounders

© 2019 Villamor-Martinez E et al. *JAMA Network Open.*

Lardon-Fernandez 2017	4	1	3	8	No adjustment for confounders
Lau 2005	4	1	3	8	No adjustment for confounders
Lee Hyun Ju 2011	4	1	3	8	No adjustment for confounders
Lee Yeri 2015	4	1	3	8	No adjustment for confounders
Leroy 2018	4	1	3	8	No adjustment for confounders
Li 2015	2	1	3	6	No CA definition, No adjustment for confounders
Lin 2005	4	1	3	8	No adjustment for confounders
Liu 2014	4	1	3	8	No adjustment for confounders
Lodha 2014	3	1	3	7	No CA definition, No adjustment for confounders
Lohmann 2014	4	1	3	8	No adjustment for confounders
Mahlman 2017	4	1	3	8	No adjustment for confounders
Mailaparambil 2010	4	1	3	8	No adjustment for confounders
May 2011	4	1	3	8	No adjustment for confounders
McGowan (h) 2009	4	1	3	8	No adjustment for confounders
Mehta 2006	4	1	3	8	No adjustment for confounders
Mestan 2010	4	1	3	8	No adjustment for confounders
Metcalfe 2017	4	2	3	9	
Miralles 2008	4	1	3	8	No adjustment for confounders
Misra 2015	4	1	3	8	No adjustment for confounders
Mittendorf 2005	4	1	3	8	No adjustment for confounders
Miyazaki 2016	4	2	3	9	
Morrow 2017	3	1	3	7	No CA definition, No adjustment for confounders
Mu 2008	4	2	2	8	BPD not defined
Nasef (h) 2013	4	1	3	8	No adjustment for confounders
Natarajan 2008	4	1	3	8	No adjustment for confounders
Nicaise 2002	3	1	3	7	Less representative population (vascular pathology excluded), No adjustment for confounders
Nishimaki 2003	4	1	3	8	No adjustment for confounders
Novitsky 2015	4	1	3	8	No adjustment for confounders
Ogunyemi 2003	4	1	3	8	No adjustment for confounders
Oh 2015	4	1	3	8	No adjustment for confounders
Ohyama 2002	4	2	3	9	
O'Shea 1998	4	1	3	8	No adjustment for confounders
Paananen 2009	4	2	3	9	
Pappas (h) 2014	4	1	3	8	No adjustment for confounders
Perrone 2012	4	1	3	8	No adjustment for confounders
Plakkal 2013	4	2	3	9	
Polam 2005	4	1	3	8	No adjustment for confounders
Prendergast 2011	4	1	3	8	No adjustment for confounders
Redline (h) 2002	4	2	3	9	
Richardson 2006	4	1	3	8	No adjustment for confounders
Rindfleisch 1996	4	1	3	8	No adjustment for confounders

© 2019 Villamor-Martinez E et al. *JAMA Network Open.*

Rocha 2006	4	2	3	9	
Rocha 2010	4	2	3	9	
Rocha 2011	4	1	3	8	No adjustment for confounders
Rojas 2012	4	1	3	8	No adjustment for confounders
Sampath 2015	4	1	3	8	No adjustment for confounders
Sato 2011	4	1	3	8	No adjustment for confounders
Schena 2015	4	1	3	8	No adjustment for confounders
Schlapbach 2010	4	1	3	8	No adjustment for confounders
Seliga-Sizecka 2013	4	1	3	8	No adjustment for confounders
Serenius 2004	3	1	3	7	No CA definition, No adjustment for confounders
Shima 2011	4	1	3	8	No adjustment for confounders
Shima 2013	4	1	3	8	No adjustment for confounders
Smit 2015	4	1	3	8	No adjustment for confounders
Soliman 2017	4	1	3	8	No adjustment for confounders
Soraisham 2009	4	2	3	9	
Soraisham 2013	4	1	3	8	No adjustment for confounders
Stepan 2016	4	1	3	8	No adjustment for confounders
Stichel 2011	4	1	3	8	No adjustment for confounders
Stimac 2014	4	1	3	8	No adjustment for confounders
Streubel 2008	4	1	3	8	No adjustment for confounders
Strunk 2012	4	1	3	8	No adjustment for confounders
Suppiej 2009	4	1	2	7	No adjustment for confounders, Loss to follow-up
Thomas 2009	4	1	3	8	No adjustment for confounders
Tokuriki 2015	3	1	3	7	No CA definition, No adjustment for confounders
Torchin 2017	4	2	3	9	
Trevisanuto 2013	4	1	3	8	No adjustment for confounders
Tsiartas 2013	4	1	3	8	No adjustment for confounders
Van Marter 2002	4	2	3	9	
van Vliet 2012	4	2	3	9	
Viscardi 2004	4	2	3	9	
Wang 2014	4	1	3	8	No adjustment for confounders
Watterberg 1996	4	1	3	8	No adjustment for confounders
Watterberg 1999	4	1	3	8	No adjustment for confounders
Watterberg 2004	4	2	3	9	
Wirbelauer 2011	4	1	3	8	No adjustment for confounders
Xie 2015	4	1	2	7	No adjustment for confounders, BPD not defined
Xie 2016	4	1	3	8	No adjustment for confounders
Yoon 1999	4	1	3	8	No adjustment for confounders
Young 2005	4	1	3	8	No adjustment for confounders
Zanardo 2002	4	1	3	8	No adjustment for confounders
Zanardo 2008	4	1	3	8	No adjustment for confounders
Zhang 2011	4	1	3	8	No adjustment for confounders

BPD: bronchopulmonary dysplasia; CA: chorioamnionitis; SGA: small for gestational age.

© 2019 Villamor-Martinez E et al. *JAMA Network Open.*
